# Structure and function of a dual antagonist of the human growth hormone and prolactin receptors with site-specific PEG conjugates

**DOI:** 10.1016/j.jbc.2023.105030

**Published:** 2023-07-11

**Authors:** Reetobrata Basu, Rich Brody, Uday Sandbhor, Prateek Kulkarni, Emily Davis, Deborah Swegan, Lydia J. Caggiano, Edward Brenya, Sebastian Neggers, John J. Kopchick

**Affiliations:** 1 Edison Biotechnology Institute, Ohio University, Athens, Ohio, USA; 2 Infinix Bio LLC, Columbus, Ohio, USA; 3 Molecular and Cellular Biology Program, Ohio University, Athens, Ohio, USA; 4 Department of Biological Sciences, Ohio University, Athens, Ohio, USA; 5 Honors Tutorial College, Ohio University, Athens, Ohio, USA; 6 Department of Medicine, Endocrinology, Erasmus Medical Centre, Rotterdam, Netherlands; 7 Heritage College of Osteopathic Medicine, Ohio University, Athens, Ohio, USA

**Keywords:** human growth hormone (hGH), hGH receptor (hGHR), hPRL, hPRLR hGHR antagonist, pegylation, anti-cancer

## Abstract

Human growth hormone (hGH) is a pituitary-derived endocrine protein that regulates several critical postnatal physiologic processes including growth, organ development, and metabolism. Following adulthood, GH is also a regulator of multiple pathologies like fibrosis, cancer, and diabetes. Therefore, there is a significant pharmaceutical interest in developing antagonists of hGH action. Currently, there is a single FDA-approved antagonist of the hGH receptor (hGHR) prescribed for treating patients with acromegaly and discovered in our laboratory almost 3 decades ago. Here, we present the first data on the structure and function of a new set of protein antagonists with the full range of hGH actions—dual antagonists of hGH binding to the GHR as well as that of hGH binding to the prolactin receptor. We describe the site-specific PEG conjugation, purification, and subsequent characterization using MALDI-TOF, size-exclusion chromatography, thermostability, and biochemical activity in terms of ELISA-based binding affinities with GHR and prolactin receptor. Moreover, these novel hGHR antagonists display distinct antagonism of GH-induced GHR intracellular signaling *in vitro* and marked reduction in hepatic insulin-like growth factor 1 output *in vivo*. Lastly, we observed potent anticancer biological efficacies of these novel hGHR antagonists against human cancer cell lines. In conclusion, we propose that these new GHR antagonists have potential for development towards multiple clinical applications related to GH-associated pathologies.

Human growth hormone (hGH) is an anterior pituitary-derived endocrine protein that exerts a range of physiological effects in the body, following binding to and activation of its cognate pre-formed hGH receptor (hGHR) homodimer expressed differentially across a wide range of cellular types in different tissues (1). The major processes regulated by the hGH–hGHR interaction includes driving postnatal somatic growth, organ development, directing the hepatic production of >75% of the circulating insulin-like growth factor 1 (IGF1), body composition, and metabolic homeostasis (anabolic to proteins and carbohydrates, catabolic to lipids) (2). Pathologically, an excess of serum hGH due to a hypersecreting pituitary adenoma causes acromegaly (3), while a subnormal production of pituitary hGH causes growth hormone (GH) deficiency and inactivating mutations of the growth hormone receptor (GHR) leads to the states of GHR resistance/insensitivity termed Laron syndrome (LS) (4).

In a normal state, there is a progressive decline in the pituitary output of hGH following adulthood, termed ‘somatopause’ (5) while, interestingly, the local, extra-pituitary production of GH with autocrine/paracrine action in specific tissues continues throughout life (6) and often increases with age (7). Importantly, congenital suppression of GH action has been found to be protective against incidence of several age-associated comorbidities such as cancer, diabetes, cardiomyopathy, glomerulonephritis, and cognitive decline in several mouse models (Ames, Snell, lit/lit, GHR−/−, GH−/−, and more) (8, 9). Remarkably, in Israel, a relatively large cohort of individuals with LS, studied for over 50 years, presented zero cases of cancer (10, 11), while another large Ecuadorian cohort of patients with LS studied for over 30 years have also presented no cases of malignancy and are resistant to diabetes (12). Moreover, adult-onset suppression of GH action in mouse models (1.5mGHRKO, 6mGHRKO) has been recently reported to recapitulate several of the aforementioned health benefits (13). Therefore, GH occupies a central position in the research on lifespan and healthspan, resulting in a significant interest in the development of pharmaceutical inhibitors of the GH–GHR interaction and subsequent intracellular signaling (14).

The GH–GHR binding is asymmetric (1:2 stoichiometry), wherein one GH molecule binds to a preformed GHR homodimer. Accordingly, the GH molecule has two distinct sites of interaction with the receptor—a high affinity–binding site 1 comprising amino acids in helices I and IV and a low affinity–binding site 2 comprising amino acids in helix III (15). Currently, the most successful way of interfering with the GH–GHR interaction is an hGHR antagonist, Somavert (pegvisomant for injection; Pfizer Inc), which was discovered in our laboratory in the early 1990s and is currently an Food and Drug Administration (FDA)-approved treatment for patients with acromegaly (16). The peptide core of the pegvisomant molecule, termed B2036, is the 191 amino acid hGH molecule with nine different amino acid substitutions of which one (G120K) makes it an hGHR antagonist by disrupting proper site 2 binding, while the other eight substitutions (H18D, H21N, R167N, K168A, D171S, K172R, E174S, I179T) impart improved affinity for site 1 binding and increased specificity for the hGHR dimer (16, 17, 18). Importantly, the human GH (but no other species GH) is known to bind to and activate the prolactin receptor (PRLR) where it exerts a lactogenic effect (19, 20, 21). The G120K-modified GH (hGH-G120K) also binds to the PRLR, unlike B2036 or pegvisomant which binds exclusively to the hGHR (22).

The versatile and pleiotropic role of hGH in normal growth and development in the early stage of life and in promoting multiple pathophysiology in mature adult life makes it an attractive pharmaceutical candidate (1, 23). The bulk of the efforts at synthesizing an active GH molecule and extending its short circulating half-life of ∼20-min involved various strategies of pegylation to treat congenital deficiencies in GH production (24). In these cases, site-specific pegylations leading to increased serum half-lives of hGH were performed by Cox *et al* generating T3C and S144C variants of the hGH (https://pubchem.ncbi.nlm.nih.gov/patent/NZ-513077-A) and by Cho *et al.* (25) generating 20 different hGH variants pegylated site specifically at six different conjugation sites (Y35, F92, Q131, R134, Y143, K143). As of now, there has been a major advancement in long-acting forms of hGH with several new approaches which have won regulatory board approvals recently. The notable candidates which have achieved an efficacious once/week administration include but are not limited to lonapegsomatropin (unmodified hGH bound to a PEG carrier molecule with a pH and temperature-dependent cleavable linker; Ascendis; approved by FDA in 2021), somapacitan (modified GH noncovalently attached to albumin; Novo Nordisk; approved by FDA in 2020), and Jintrolong (hGH attached to a 40-kDa PEG; GeneScience; approved and marketed in China) (26). On the other hand, to extend the half-life of the hGHR antagonist B2036 in circulation for therapeutic viability, four to six molecules of linear 5-kDa amine-reactive PEGs are nonspecifically covalently attached to internal lysine residues and the *N*-terminus of the first amino acid, phenylalanine, resulting in the final pegylated molecule—pegvisomant—with a mean half-life of ∼70-h in humans (16, 17, 18). Pegvisomant is a competitive inhibitor of the hGHR and in clinical trials was >90% effective in normalizing serum IGF1 in patients with acromegaly, despite a >20-fold reduction in hGHR-binding affinity compared to hGH or the hGH-G120K (27).

There remains considerable room for improvement in designing a new novel long-acting hGHR antagonist, given that pegvisomant does present an end product heterogeneity given the variable number of PEGs attached per peptide, as well as the amino acid residues to which they are attached. Additionally, the pegylations drastically reduce the binding affinity of the hGHR antagonist to the hGHR to less than 5% of the original ligand hGH. Thereafter, a number of subsequent efforts at developing improved hGHR antagonists have focused on improving the pegylation pattern in the original peptide core—B2036—of the pegvisomant molecule. In this regard, Perry and Maynard have generated three successive new designs of the modified GH molecule. First, the group generated an hGH-G120R or B2036 with *N*-terminal thioredoxin fusion and conjugated to multiple amine-reactive 5-kDa methoxy PEG succinimidyl propionate, which had a >15-h circulating half-life in mice and improved *in vitro* bioactivity than pegvisomant, albeit a significant end product heterogeneity (28). The two other antagonist designs from this group involved site-specific pegylations: (i) replacing tyrosine-35 with an unnatural amino acid propargyl tyrosine to which 5, 10, or 20 kDa azide-containing PEGs were attached by Cu-catalyzed click reactions (29) and (ii) replacing serine-144 by cysteine to which 20, 30, or 40 kDa methoxy-PEG maleimide were attached (30). Both modifications improved serum half-life and bioactivity of the core B2036 molecule. Other attempts of an *N*-terminal site-specific pegylation of hGH-G120R remained unsuccessful, while a dimeric hGH-G120R generated by *N*-term to C-terminal fusion yielded an agonist in place of an antagonist (31). The only other study which performed successful *N*-terminal mono-pegylation of B2036 with either a 20-kDa or a 40-kDa PEG reported complete loss of antagonist activity by the larger PEG, likely from steric hindrance in binding to the hGHR (32).

Although promising as hGHR antagonists, none of these molecules are known to inhibit the binding of the GH to the PRLR with an affinity similar to that of prolactin. Therefore, it is apparent that there is a distinct viable space for the development of a dual antagonist of the hGH–GHR and hGH–PRLR interactions by harnessing the ability of the G120K-hGH molecule to bind to and inhibit hGH binding to both of these receptors, unlike the GHR-specific B2036 molecule. Using site-specific pegylations (33, 34), there is the opportunity to add unique large or charged PEGs to G120K-hGH to effectively increase half-life without reducing receptor affinity, allowing a putatively reduced dosing frequency.

We aimed to develop a ‘better’ antagonist of the full spectrum of hGH action, targeting both the hGH–GHR and hGH–PRLR interactions, with a site-specific and reduced number of unique pegylations towards maximally preserving the GH-binding affinity of the GHR antagonist hGH-G120K. Previously, we reported the design and preparation of multiple analogs of hGH-G120K where one or two specific amino acids were substituted with cysteine at positions distal to the GH-GHR–binding interface (31), as determined from the X-ray structure of hGH bound to a receptor dimer (25, 26) and the very similar structure of hGH-G120R bound to a receptor monomer (35). To these substituted cysteines, maleimide PEGs were specifically conjugated to build a library of multiple putative hGHR antagonists. GHR-binding studies resulted in the selection of amino acids T142 and H151 as the positions in hGH-G120K whose substitution with cysteine and subsequent pegylation caused the least reduction in receptor-binding activity (31).

In terms of characterizing the binding efficiencies of hGHR antagonists to their target (GHR), the unmodified hGH-G120K was found by Ross *et al.* (36) to inhibit the binding of I^125^ GH to GHR on the surface of cells with a Ki of 4.7 nM, while hGH inhibited with a Ki of 3.5 nM. The Ki values for hGH and hGH-G120K inhibition of I^125^ GH binding to the extracellular domain of GHR (GHBP) were 2.3 nM and 2.6 nM, respectively. In this paper, we used a competitive ELISA to determine the relative abilities of hGH and the pegylated hGH-G120K mutants to inhibit the binding of biotin-hGH to the soluble domain of the hGHR immobilized on an ELISA plate.

The biological assays described in this paper were performed with two types of GHR antagonists that were pegylated on selected amino acid positions; hGH-G120K that contains a single substituted cysteine (T142C) that is conjugated to a 40 kDa 2-branched PEG (GL2-400) and hGH-G120K that contains two cysteine substitutions (T142C; H151C) which are each conjugated to a 4.5 kDa tribranched PEG (dPEGA) that has an anionic carboxyl group at the terminus of each branch (Table 1). Both types of pegylated hGHR antagonists showed robust inhibition of GH-induced downstream signaling and GH-driven oncogenic processes in cultured human cancer cell lines.

**Table 1:** Abbreviations for GHRAs used in this study

Abbreviation	GHR antagonist
Compound G	GGSSG-hGH-G120K-T142C-dPEGA-H151C-dPEGA
Compound G’	M-hGH-G120K-T142C- dPEGA-H151C-dPEGA
Compound D	GGSSG-hGH-G120K-T142C-GL2-400

The 2-branched 40 kDa PEG was chosen because of its proven efficacy in increasing proteins’ half-lives. Substituting this molecule on the N terminus (37) or C terminus (38) of GH or substituting a linear 40 kDa PEG on the N terminus on the hGHR antagonist B2036 (39) resulted in greatly extended *in vivo* half-lives although the losses in binding activities were significant. The 4.5 kDa tribranched anionic PEG was chosen for conjugation to two specific hGHR antagonist sites to produce a smaller hGH antagonist with the potential to have greater tumor penetration than the antagonist with the larger 40 kDa PEG (40). We previously showed that the half-life of a 50 kDa protein conjugated to one 4.5 kDa tribranched anionic PEG was ∼6 h compared with a half-life of ∼2 h for the same protein conjugated to a 4.5 kDa neutral analog (34). In this paper, we describe the structure and function of these three novel hGHR antagonists.

## Results

### Preparation of pegylated hGHR antagonists

In this study, *Escherichia coli* cells that expressed an hGHR antagonist were disrupted by sonication and, after partial purification, any non-native disulfides were reduced by the addition of low concentrations of reduced glutathione. In the case of the His-tagged compound G and compound D precursors, the glutathione reduction was done concomitantly with the TEV cleavage. The glutathione was removed by size exclusion chromatography (SEC) prior to pegylation in all cases. There was considerable variability in the oxidation state of the native disulfides after glutathione treatment, as detected by small scale pegylation reactions. In some cases, SDS-PAGE analysis showed that the number of added PEGs was equal to the number of substituted cysteines, indicating that the native disulfides had formed. In other cases, the product was a mixture of multiple PEG conjugates with higher molecular weights, indicating that some of the native cysteines did not form disulfides but instead reacted with the PEG-maleimides. To remedy the variability in the extent of thiol oxidation, small scale pegylations were performed after the glutathione removal step. If SDS-PAGE analysis of the small-scale reaction indicated that higher molecular weight species formed, then the native thiols were oxidized by the addition of copper (33, 41). After copper oxidation, the reaction was again monitored by small scale pegylation and SDS-PAGE. Large scale pegylation was performed only after higher molecular weight species were no longer detected in the small-scale reactions, indicating that the native disulfides had formed.

### Characterization of pegylated hGHR antagonists

#### Sodium dodecyl sulfate-polyacrylamide gel electrophoresis

**Figure 1:**
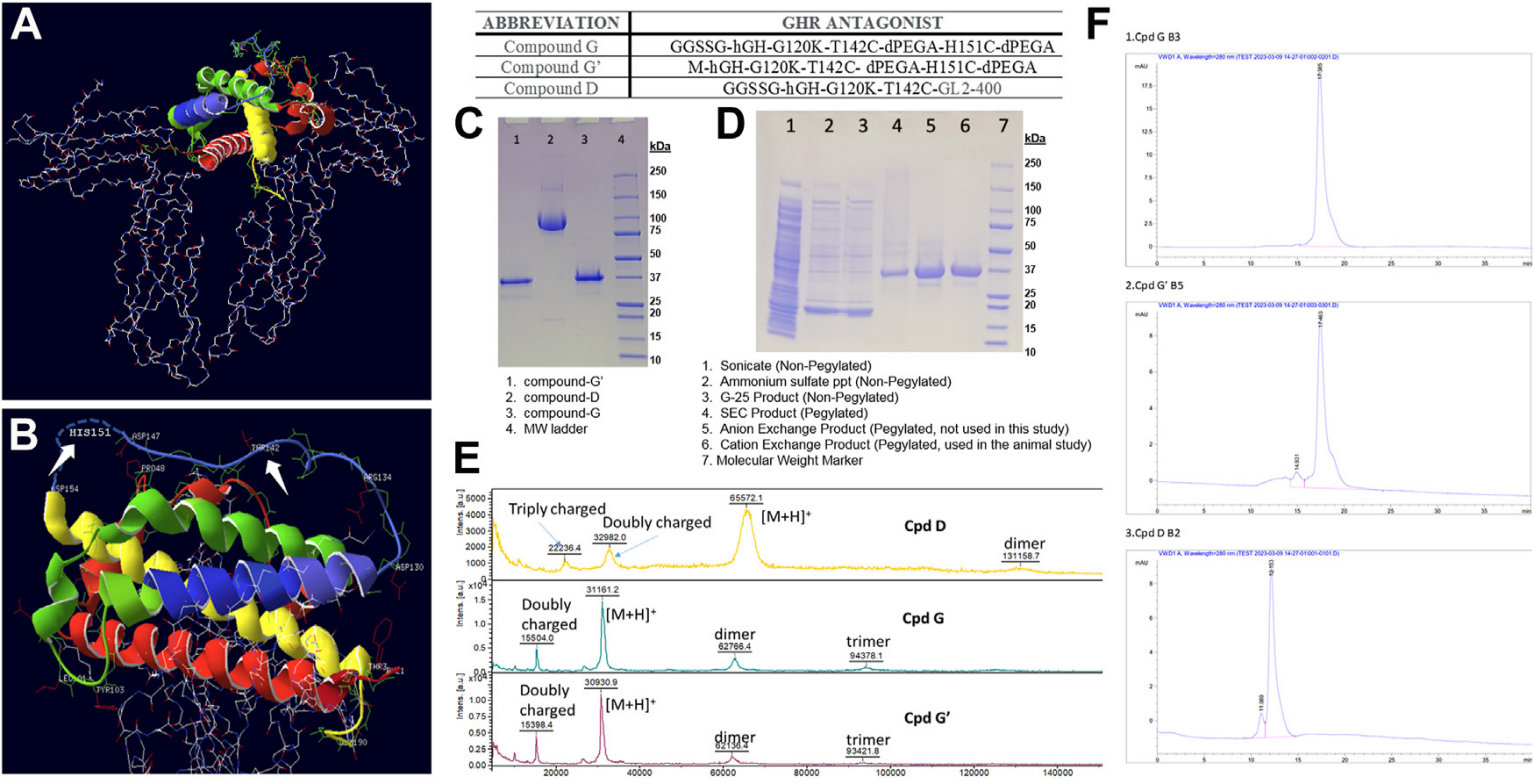
**Characterization of pegylated GHR antagonists.***A*, crystal structure of hGH (ribbon) bound to hGHR dimer (*stick*). *B*, *white arrows* indicating Thr142 and His151 residues in hGH amino acids facing away from the GH-GHR–binding site chosen based on solvent accessibility and steric access, for cysteine substitutions and subsequent site-specific PEG attachments. *C*, SDS-PAGE gel of purified GHR antagonists. *D*, SDS-PAGE gel after the different steps of the compound G′ preparation. *E*, MALDI-TOF mass spectra of compound D, compound G, and compound G’. *F*, analytical SEC profile of compounds G, G′, and D (*top* to *bottom*). hGH, human growth hormone; hGHR, hGH receptor; SEC, size-exclusion chromatography.

The SDS-PAGE gel of the hGHR antagonists is shown in Figure 1*C*. There is a single major band for each antagonist at an apparent molecular weight higher than the calculated MW. However, pegylated proteins have been shown to have lower than expected mobilities during electrophoresis (42). There are also a number of fainter bands that can be seen in each lane. The gel was analyzed by ImageJ to determine purity of the hGHR antagonists (43). The major product bands for compound G, compound D, and compound G′ comprised 92%, 89%, and 87% of the total protein respectively for each antagonist. No impurity band accounted for more than 6% of the totals. Figure 1*D* shows the SDS-PAGE bands at each step of the compound G′ purification. The effect of the addition of the two molecules of dPEGA to hGH-G120K-T142C-H151C can be seen by comparing the bands in lanes 3 and 4.

#### Matrix-assisted laser desorption ionization time-of-flight

The MALDI-TOF mass spectra of Compounds D, G, and G’ (Fig. 1*E*) all show a single major [M + H]^+^ peak. The MALDI-TOF experimental [M + H]^+^/calculated [M + H]^+^ values are as follows: compound D, 65.6 kDa/62.5 kDa; compound G, 31.2 kDa/31.5 kDa; compound G′, 30.9 kDa/31.2 kDa. This data shows that the expected number of PEG molecules are conjugated to each antagonist. The large difference between the experimental and calculated molecular weights for compound D is due to the polydisperse nature of the 40 kDa PEG, resulting in a broad MALDI-TOF peak.

#### Analytical SEC

The SEC traces for the three pegylated hGHR antagonists are shown in Figure 1*F*. Compound G shows a single major peak with a trailing shoulder and no significantly higher molecular weight peaks. Compound G′ shows a single large peak with a trailing shoulder and some small higher molecular weight peaks. Compound D shows a large peak that accounts for 90% of the total peak area and a higher molecular weight peak that accounts for the remaining 10% of the peak area.

#### Stability

The ability of the three hGHR antagonists and hGH to inhibit the binding of biotin-hGH to immobilized hGHR after three freeze-thaw cycles and after incubation overnight at 4 °C and 24 °C is shown in Table S1. The three GHA antagonists retained over 80% of their activity after three freeze-thaw cycles and retained over 89% of this activity after overnight incubation at both 4 °C and 24 °C. These results indicate that the pegylated antagonists are generally stable and are expected to perform well in the receptor-binding assays that are performed with single-use frozen aliquots and where samples are only subject to 4 °C and room temperature (RT) incubations for short periods of time. hGH was similarly stable at 4 °C and 24 °C but retained only ∼70% of its activity after a single freeze-thaw cycle.

### Relative affinities of the pegylated antagonists for the hGHR

The hGHR antagonists were designed so that the conjugated PEG molecules would not interfere with receptor binding (31) with the expectation that the binding constants for the pegylated antagonists and hGH would be similar. A competitive ELISA assay was used to determine the IC50 values for the inhibition of biotin-hGH binding to the soluble domain of the GHR immobilized on an ELISA plate. The ratio of the IC50 value for hGH to the IC50 value for each antagonist is equal to the relative binding affinities of that antagonist compared with hGH.

**Figure 2:**
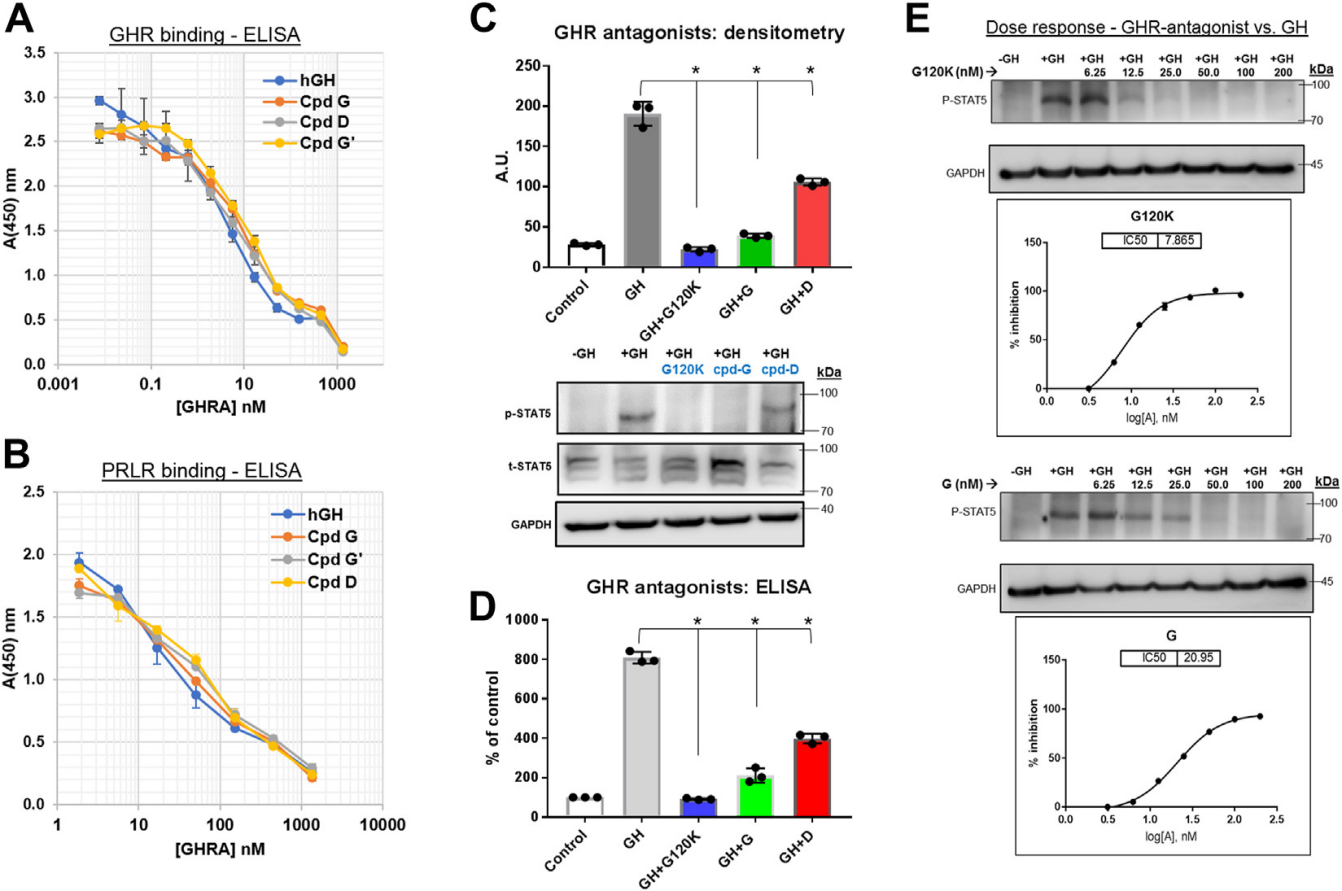
**Receptor binding and inhibition of GHR antagonists.***A*, competitive ELISA assay for the ability of the GHR antagonists (GHRA) to inhibit the binding of biotin-hGH to soluble GHR immobilized on an ELISA plate. *B*, competitive ELISA assay for the ability of the GHR antagonists to inhibit the binding of biotin-hGH to soluble PRLR immobilized on an ELISA plate. *C*, successful binding of GH to GHR leads to downstream activation of STAT5 by phosphorylation at residues 694 and 699 by GHR-associated JAK2. This phosphorylation of STAT5 is a hallmark of GHR signaling and is used as a surrogate assay for GHR activation/inhibition. *C*, Western blot and (*D*) competitive ELISA analysis of inhibitory effects of 200 nM of compounds (unpegylated hGH-G120K, compound-D, or compound-G in STAT5 phosphorylation of MALME-3M cells treated with 2.5 nM (∼50 ng/ml) human GH. Tukey’s multiple comparison test was done for (*C*) and (*D*) using GraphPad Prism(v7) and ∗ represents *p* < 0.001. *E*, Western blot analysis of STAT5 phosphorylation following treatment with increasing doses (6.25–200 nM) of GHR antagonists *versus* 2.5 nM hGH. EC50 of compounds G120K and compound-G in inhibiting hGH induced STAT5 phosphorylation in GHR-rich human melanoma cells MALME-3M. hGH, human growth hormone; PRLR, prolactin receptor.

Figure 2*A* shows the results of competitive inhibition assay, and Table 2 shows the IC50 values from four independent competitive assays. Using the IC50 hGH/IC50 antagonist ratio, compounds G, D, and G′ are calculated to retain 70%, 90%, and 40% of hGH’s affinity for the receptor, respectively. The result for compound D, which is conjugated to a 40 kDa 2-branched PEG at position 142 and retains 90% of hGH’s binding activity, can be compared to the 5% retention of binding activity when hGH is substituted with a 2-branched 40 kDa PEG on its N terminus (37) or the 1% retention of binding activity when hGH is substituted with the same PEG on its C terminus (38). As per the *t* test, the IC50s for compounds G and D are not significantly higher than the IC50 for hGH (*p* > 0.05; one tailed test). In contrast, the IC50 for compound G′, which has 40% of the hGH’s affinity for the receptor, is significantly higher than the IC50 for hGH (*p* < 0.05; one tailed test). Considering the sequenced similarity of compounds G and G′ and the fact that, by SDS-PAGE (Fig. 1), both compounds are substantially pure, it is likely that the lower binding activity of compound G′ is due to misfolded protein due to different preparation procedures.

**Table 2:** Inhibition of the binding of Biotin-hGH to immobilized GHR^a^

Inhibitor	IC50 nM				Average IC50, nM	Relative affinity for immobilized GHR (IC50 hGH/IC50 GHA)
	Run 1	Run 2	Run 3	Run 4		
hGH	9.1	5.4	8.1	5.8	7 ± 2	1 (defined)
Cpd G	15.9	4.5	10	11	10 ± 5	0.7
Cpd D	9.4	3.6	9.5	8.6	8 ± 3	0.9
Cpd G’	15.9	14.8	21.2	14.1	17 ± 3	0.4

a The IC50 entries in this table are average values ± 1 STDEV from four independent assays (Runs 1–4) of the type shown in Figure 2.

### Relative affinities of pegylated antagonists for the hPRLR

A single competition ELSA was performed to determine whether the three GHR antagonists, like hGH, bind to the hPRLR. Figure 2*B* clearly shows that these antagonists, unlike pegvisomant (20), bind to the hPRLR with affinities similar to that for hGH.

### *In vitro* evaluation of hGHR antagonism by compound G and compound D

Tyrosine phosphorylation of STAT5, the hallmark surrogate assay for hGHR activation, was evaluated using hGHR-rich human melanoma cells, and employing both western blots and ELISA showed that the efficacy of the MAL-dPEG-A conjugate of double cysteine–substituted GGSSG-hGH-G120K (compound G) was comparable to the unpegylated GGSSG-hGH-G120K and suppressed STAT5 phosphorylation by >90% (Fig. 2, *C*–*E*). The polydisperse GL2-400 conjugate of a single cysteine–substituted GGSSG-hGH-G120K, (compound D) suppressed cellular STAT5 phosphorylation by 46%. Comparing increasing doses (6.25–200 nM) of compounds D and G in the cell-based assay, compound G was found to be more efficacious (EC50 = 20.9 nM) than compound D (EC50 >100 nM) and close to the unpegylated GGSSG-hGH-G120K (compound A, EC50 = 7.9 nM) in inhibiting hGH-induced STAT5 phosphorylation in human melanoma cells (Fig. 2*E*). Moreover, comparing increasing doses of hGH (0.25–200 nM) against 50 nM of compounds A or D or G, compound G was found to be as efficacious as unpegylated GGSSG-hGH-G120K in inhibiting hGH binding (Fig. S3). Further evaluation of *in vivo* efficacy in lowering circulating IGF1 levels in mice were performed using purified compound G.

### *In vivo* efficacy of hGHR antagonist compound G′

**Figure 3:**
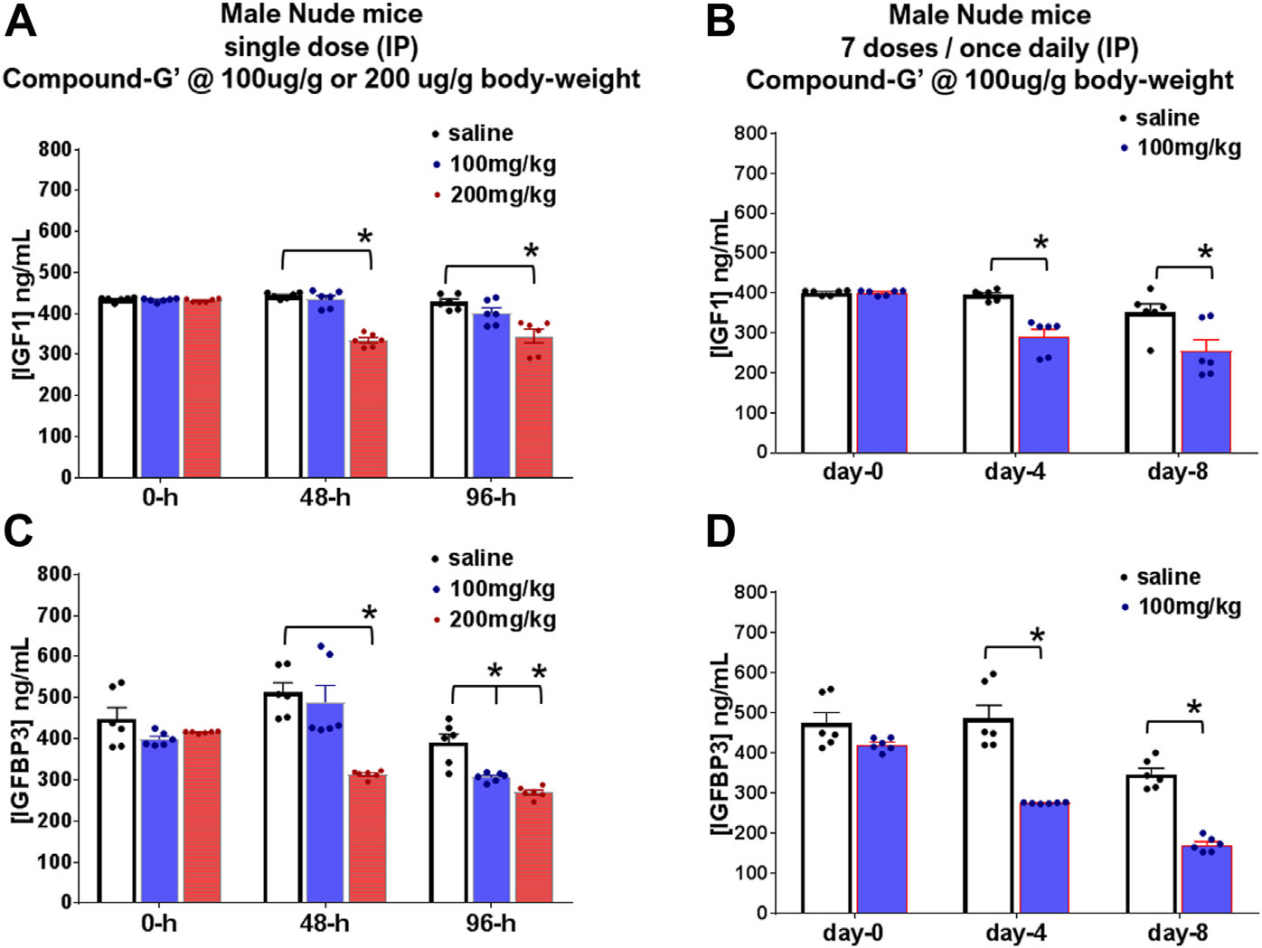
**Suppression of GH-target genes IGF1 and IGFBP3 levels by compound-G′ in mice.** 3-months-old male Nude mice (n = 4) were intraperitoneally injected with (*A* and *B*) either a single intraperitoneally (i.p.) injection of compound-G′ at either 100 or 200 mg/kg body weight or PBS (control), and serum was collected 48 and 96 h of the injection or (*C* and *D*) i.p. injected with 100 mg/kg body weight of compound-G′ once daily (intraperitoneal injection) for 7 days and serum was collected on day 4 and day 8 1 h after of the injections. Commercial kits for circulating levels of IGF1 (*A* and *C*) and IGFBP3 (*B* and *D*) were used as per manufacturers protocol. (∗ *p* < 0.01, Bonferroni’s multiple comparisons test, n = 6). IGF1, insulin-like growth factor 1; IGFBP3, IGF1-binding protein 3.

A principal physiologic action of hGH is the hepatic production of IGF1 contributing as much as 75% of the circulating IGF1. Therefore, the lowering of circulating IGF1 by the hGHR antagonist pegvisomant is a robust surrogate biomarker of its *in vivo* efficacy. However, pegvisomant, although is an effective antagonist of the hGHR, does not act as a strong antagonist of the mouse GHR, requiring high doses to cause significant observable effects. Firstly, we observed that in cultured mouse bladder cancer cells and mouse fibroblast cells, while compound D was comparable to pegvisomant in suppressing bGH-induced STAT5 phosphorylation downstream of GHR activation, compound G exhibited a clear and profoundly superior GHR inhibition than either pegvisomant or compound D, by almost completely inhibiting the downstream STAT5 phosphorylation (Fig. S4). Next, we treated Nude male mice with compound G′ in two different regimens—either a single i.p. injection of either 100 or 200 mg/kg body weight or seven consecutive once daily i.p. injections of 100 mg/kg body weight. We observed that unlike a single dose of 100 mg/kg compound G′ which did not decrease circulating IGF1, a single 200 mg/kg dose suppressed circulating IGF1 by 24% at 48-h and by 19% at 96-h postinjection (Fig. 3*A*). Corresponding decreases in circulating IGF1-binding protein 3 (IGFBP3), another hepatic biomarker of GH action, were suppressed by 38% at 48-h and by 31% at 96-h postinjection in the same mice (Fig. 3*B*). On the other hand, seven once daily i.p. injections of 100 mg/kg compound G′ suppressed IGF1 levels by 26% at day 4 and 27% at day 8 of treatment (Fig. 3*C*). Compound G′ also suppressed circulating IGFBP3 levels at day 4 and day 8 in the same mice significantly by 43% and 50%, respectively (Fig. 3*D*).

### Anticancer bioactivity of compound G in human cancer cells

**Figure 4:**
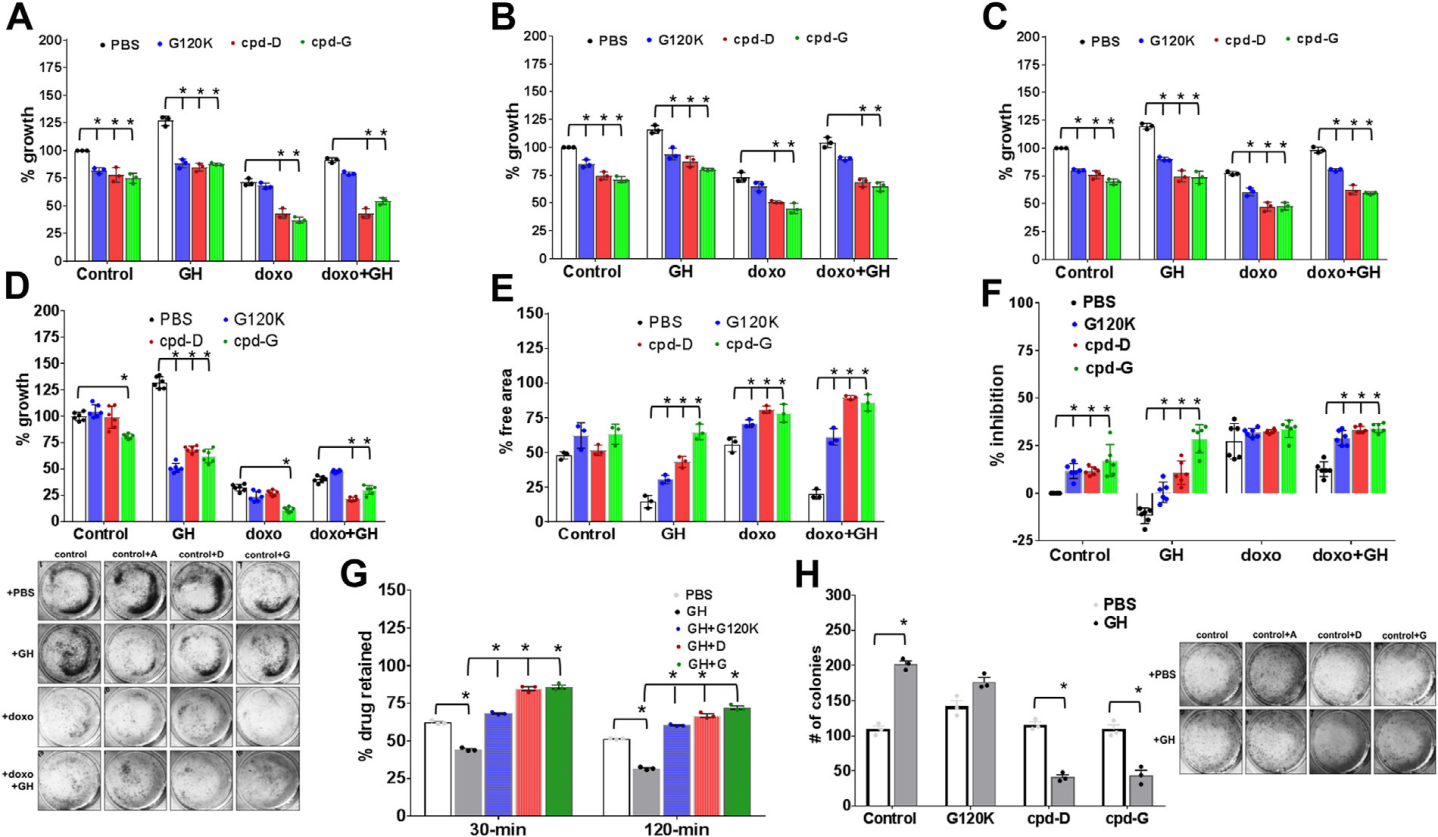
**Anticancer efficacy of GHR antagonists.** The unpegylated GGSSG-hGH-G120K, compound-D, and compound-G were tested for their anticancer properties by virtue of their GH inhibitory effects. *A*–*C*, cell viability of human melanoma cells MALME-3M (*A*), SK-Mel-28 (*B*), SK-Mel-30 (*C*) following 72-h treatments with either saline (control) or 5 nM hGH or 200 nM doxorubicin or doxorubicin and hGH in either presence or absence of saline or G120K or compound-D or compound-G at 500 nM was assessed using resazurin cell viability assay. *D*, similar 72-h treatments followed by crystal violet staining shows number of viable colonies on the plate. *E*–*H*, the efficacy of G120K or compound-D or compound-G in inhibiting MALME-3M migration rate (*E*), basement membrane invasion rate (*F*), DiOC2 (dye, ABC transporter substrate, and surrogate for chemotherapy) retention (*G*), and postchemotherapy colony formation (*H*). All experiments were done at least three times (n = 3, ∗*p* < 0.01, Tukey’s multiple comparisons test). hGH, human growth hormone.

Human melanoma cells express a consistently high level of hGHR (44) and are highly responsive to hGH action, which regulates multiple oncogenic pathways and processes in this cancer (45, 46, 47, 48, 49, 50). Therefore, a GHR antagonist is a candidate negative regulator of melanoma cell growth and therapeutic response. Herein, we compared our pegylated compounds D and G against the unpegylated GGSSG-hGH-G120K (referred to here as ‘G120K’) in human melanoma cells. Exogenous hGH addition significantly increases cell viability over a 72-h period including a rescue from the cytotoxic effects of the chemotherapy doxorubicin in three different melanoma cell lines MALME-3M, SK-Mel-28, and SK-Mel-30 (Fig. 4, *A*–*C*). Compounds D and G markedly suppressed this effect of hGH across all three cell lines and significantly improved the therapeutic efficacy of doxorubicin as observed by resazurin assay (Fig. 4, *A*–*C*, Supplemental Excel file) and posttreatment crystal violet staining of viable colonies (Fig. 4*D*). In most cases, the compounds D and G had better antagonist activity over the 72-h period compared to the unpegylated counterpart (hGH-G120K), while there was no consistent significant difference in effects between the two pegylated compounds.

We and others have previously shown that hGH action in melanoma upregulates the epithelial-to-mesenchymal transition program (*via* upregulation of expression of epithelial-to-mesenchymal transition–related transcription factors) in these cancer cells, thereby promoting their migration and invasion potential (45, 46, 47, 48, 49, 50). Accordingly, the cell migration rate in the absence and presence of hGH was evaluated over a 48-h period including treatments with doxorubicin alone or in conjunction with the candidate GHR antagonists. Both pegylated compounds D and G as well as the unpegylated G120K significantly suppressed melanoma cell migration (increased % area free from migrating cancer cells), more pronouncedly in combination with doxorubicin (Figs. 4*E* and S5, Supplemental Excel file). A similar effect was seen in the basement membrane invasion assays over a 48-h period, wherein both pegylated compounds D and G as well as unpegylated G120K inhibited the invasive effects of GH and improved the cytotoxicity of doxorubicin treatment (Fig. 4*F*, Supplemental Excel file). These inhibitory effects of compounds D and G on melanoma cell invasion was further corroborated by evaluating viable colony-forming abilities of detached cells in the supernatant, following doxorubicin treatment in the presence or absence of hGH. Over a 14-day period, compounds D and G but not the unpegylated G120K effected a 3- to 4-fold decrease in the number of viable colonies, as observed by crystal violet staining (Fig. 4*H*). Moreover, hGH action is known to drive drug resistance in human melanoma cells by upregulating the expression of multidrug efflux pumps—ABC transporters which allows lower chemotherapeutic retention in the tumor cells, thus enabling chemoresistance (48). One week pretreatment of melanoma cells with GH and/or GHR antagonists, followed by drug-retention assay loading DiOC_2_ (3) as a drug surrogate, showed a 1.5- to 2-fold increase in the amount of dye retained within the cells over a 2-h period by compounds D and G compared to the hGH-induced efflux (Fig. 4*G*). Altogether, both compound D and compound G exhibit potent anticancer properties, as a function of inhibiting hGH action, in human melanoma cell lines. Additionally, compound G also strongly inhibited the growth-promoting effects of hGH and improved doxorubicin cytotoxic effects in human breast cancer cell lines T47D and MDA-MB-231. Both these cell lines have a much higher hPRLR:hGHR ratio, compared to the human melanoma cells (Fig. S6). The abrogation of hGH-regulated hGHR and hPRLR activation is particularly relevant for therapeutic approaches against breast and prostate cancers. This fact is highlighted by a markedly higher hazard ratio and decreased survival in breast cancer patients (the cancer Genome Atlas data base) with higher expression signature of hGH, hGHR, and hPRLR together, compared to hGHR or hPRLR alone (Fig. S7).

## Discussion

In the current study, we describe the synthesis, purification, and characterization of biologically active dual antagonists of the hGH-induced activation of hGHR and hPRLR, through introduction of cysteine residues at specific sites that are distal to the ligand-receptor–binding interfaces and subsequent conjugation with activated PEG reagents. The purification and pegylation procedures were designed to facilitate the formation of the two native hGH disulfide linkages while keeping the two cysteines introduced at positions 142 and 151 of compounds G and G′ or the single cysteine at position 142 of compound D in the reduced state so they could be pegylated. After pegylation, purification by either SEC or SEC followed by ion exchange chromatography resulted in highly purified preparation of compounds G, D, and G’. The pegylated GHR antagonists described in this study are significantly superior in maintaining their binding affinities than pegvisomant (<5%), with compound G, compound D, and compound G′ exhibiting, respectively, binding affinities of about 70%, 90%, and 40% of the original hGH molecule. In the biological activity assays, compound G was particularly effective and is the current focus of our anticancer studies.

Attachment of PEG moieties onto therapeutic compounds, first discovered in the 1970s by Davis *et al.* (51), increases the net size, hydrodynamic radius, and the molecular weight of proteins and also alters their physicochemical properties like spatial hindrance, conformation, and electrostatic binding. Successful pegylation results in decreased systemic clearance, macrophage uptake, and proteolysis of the proteins, as well as decreased immunogenicity (52). For example, pegylation of the hGH antagonist B2036 with 4 to 6 5-kDa PEG moieties on random lysines and the *N*-terminal phenylalanine (pegvisomant) significantly decreased B2036’s binding affinity by about 20-fold but greatly increased its circulating half-life in humans to ∼70-h from 20-min (14). hGH substituted with a 2-branched 40 kDa PEG on its N terminus (37) or its C terminus (38) has been shown to also have a greatly extended *in vivo* half-life. However, the affinity for the hGHR decreased by ∼20 times after conjugation to the N terminus of hGH and by over 100 times after C-terminus conjugation. Likewise, when the hGHR antagonist B2036 (unpegylated pegvisomant) was conjugated to a linear 40 kDa PEG on its N terminus (39), the half-life was greatly extended but the binding to the hGHR was decreased ∼20 fold. In contrast, we have found that conjugating a 40 kDa 2-branched PEG to amino acid position 142 of hGH-G120K (compound D) results in almost no decrease in receptor binding. From the literature precedents described above, it is expected that compound D will have an extended *in vivo* half-life.

The 4.5 kDa tribranched discrete PEG (dPEG-A), which is a component of compounds G and G′, was chosen for three reasons. First, it was previously shown that a 50 kDa antibody fragment conjugated to a single molecule of this tri-anionic 4.5 kDa PEG had an approximate half-life of 6 h in mice compared to an approximate 2 h half-life for the same antibody fragment conjugated to the analogous neutral PEG (34). It was postulated that attaching two 4.5 kDa tribranched PEGs to the 22 kDa hGH-G120K protein would result in a similarly extended half-life. Second, large PEG molecules, such as the 40 kDa–branched PEG used to make compound D, are made by polymerization and contain a range of molecular weight molecules. The 4.5 kDa PEG, in contrast, is a single molecule “discrete PEG” made by step-by-step chemical synthesis (https://pubchem.ncbi.nlm.nih.gov/patent/NZ-513077-A). Finally, the smaller antagonist with two tri-anionic 4.5 kDa PEGs has the potential to have greater tumor penetration than the antagonist with the larger 40 kDa PEG (40).

Following the discoveries of the covert actions of hGH in driving multiple human pathologies, a major pharmaceutical interest in targeting the hGH–hGHR binding towards antagonizing hGH action ensued. The targetability of the hGHR was established by the landmark discovery of the first protein antagonist, pegvisomant, in our laboratory in the early 1990s, which was a modified hGH molecule with multiple 5-kDa nonspecific pegylations. Pegvisomant is a successful drug (marketed by Pfizer and prescribed for acromegaly) which acts as a competitive inhibitor/antagonist of endogenous hGH binding to the hGHR and normalizes serum IGF1 in >90% of the patients in clinical trials. Additionally, it has an impressive safety profile (18). However, the pegvisomant molecule displays (i) end product heterogeneity due to the nonspecific and variable (four to six) number of linear pegylations per molecule, (ii) reduced affinity to the target hGHR compared to hGH, requiring an elevated dose (10–30 mg/day), and (iii) does not inhibit the hGH–PRLR binding which can be physiologically crucial especially in the context of increased circulating hGH levels following pegvisomant treatment. In addition, the high economic burden of treatment with pegvisomant emphasizes the requirement for developing a comparably cost-effective next-in-class hGHR antagonist (39).

hGH itself can bind to and potently (as much as by prolactin itself) activate the hPRLR (20) as well as hGHR/hPRLR hybrid receptors (53). Importantly, the net amino acid modifications on pegvisomant prevents it from antagonizing the hGH-hPRLR binding (22)—a critical factor for multiple reasons as follows. Anywhere from 15 to 40% of patients with acromegaly (a condition of GH excess due to a hypersecreting pituitary adenoma) also suffer from hyperprolactinemia due to elevated secretion of hPRL from the pituitary cells (54, 55, 56). These patients with acromegaly and hyperprolactinemia (adenomas secreting both hGH and hPRL) have significantly more severe menstrual disorders, galactorrhea, poorer surgical control, and higher adenoma relapse rates than GH-only pituitary adenoma patients (55). Moreover, there are numerous reports that in specific cancers, especially breast, endometrial, liver, and prostate cancers, the hPRLR is often upregulated along with a concomitant increase in the hGHR expression (22, 55, 57, 58, 59, 60). In these cancers, either the hGHR or the hPRLR or both have been targeted by either receptor-specific antagonists or antibodies as well as by bispecific (hGHR + hPRLR) antibodies (61, 62). Therefore, the concomitant inhibition of hGHR and hPRLR activation is a promising approach in cancer therapy, especially in target scarce situations like triple-negative breast cancer. Furthermore, different hPRLR antagonists are under development for the treatment of different kinds of ‘*unresolved systemic and local hyperprolactinemia*’ (63). Lastly, therefore, the hPRLR-activating effect of hGH is crucial in all the above cases of acromegaly, cancer, and systemic/local hyperprolactinemia. The current study can potentially address these factors as we present novel antagonists of the complete hGH action, with distinct advantages. As we showed here, compared to the currently approved pegylated hGHR antagonist pegvisomant, compounds G, D, and G′ have the advantages of site specificity of pegylations, number of pegylations and hence no end product heterogeneity, and markedly higher binding affinity to the hGHR (Table S2). Additionally, unlike pegvisomant, our compounds also exhibit strong hPRLR-binding affinity which allows them to be novel candidates for a wider spectrum inhibition of hGH action. Lastly, they can be an excellent investigational tool as they appear to be superior to pegvisomant in antagonizing mouse GHR activation.

In our earlier report, we described a short peptide (16 amino acids)–S1H–as a novel antagonist of the GHR (64). By virtue of its design mimicking the residues 36 to 51 comprising the mini-helix of hGH molecule, which is also important for hGH–hPRLR binding, S1H successfully inhibited STAT5 activation following hGH as well as hPRL treatment on cells harboring both hGHR and hPRLR receptors. Therefore, our previous hGHR antagonist candidate S1H is also a dual antagonist of hGH action but markedly differs in size, composition, mechanism of action and lacks any pegylations compared to compounds G, G′, and D. Therefore, S1H compared to the compounds in our current report is also expected to have a distinct pharmacokinetic and pharmacodynamic profile and awaits empirical validation. Direct comparative studies of antagonistic properties of S1H against compound G or pegvisomant are forthcoming and not reported in this manuscript.

The marker for *in vivo* bioactivity of hGHR antagonists is the lowering of serum IGF1 and IGFBP3. Studies in rodents with pegvisomant at a variety of doses and regimen have reported differential levels of IGF1 reduction and do not perform well overall. However, pegvisomant administered (intra-peritoneal (IP)) in Nude mouse model of colorectal cancer over 30-days at 60 mg/kg lowered IGF1 by 57 to 64% (65), while a 14-days treatment (IP) at 250 mg/kg reduced IGF1 in Nude mouse model of breast cancer by 70 to 80% (66). From earlier successful pegylations of the hGHR antagonist, a 20-kDa *N*-terminal pegylated B2036 administered in rats at 2 mg/kg suppressed IGF1 by 30 to 43% (32), while Perry and Maynard’s 40-kDa S144C pegylated B2036 administered in mice at 10 mg/kg suppressed IGF1 by almost 51% (30). In our current study, we performed two sets of treatment regimens. In a single bolus of compound G at 100 mg/kg, we did not observe a significant drop in serum IGF1 or IGFBP3 at 48 or 96-h post-treatment. However, a 200 mg/kg single IP injection caused a 24% reduction in serum IGF1 and a 39% drop in serum IGFBP3 at 48-h post-treatment. A 7-days once daily injection at a dose of 100 mg/kg caused a 26% drop in 4 days and 40% drop at the end of study (day 8) in serum IGF1, with a more pronounced effect observed for serum IGFBP3. Therefore, it is apparent that compound G is a potent antagonist of GH-induced hepatic IGF1 in mice, while an empirical comparison with pegvisomant is pending. Importantly, due to a much faster rate of metabolism in mice than humans (67), the half-life of pegvisomant is significantly lower in mice (∼20-h) than in humans (∼70-h). In the current study, the pharmacokinetic studies to evaluate the *in vivo* half-life of compound G have not been performed. Future studies incorporating multiple types of pegylated hGH-G120K constructs compared against pegvisomant in mice will allow a distinct comparison, allowing room for additional chemical modifications to select the best candidate compound balanced in serum half-life, GHR binding, and *in vivo* bioactivity profiles. Additional variabilities are expected across strain and sex of the animals. These studies are currently underway.

Compound G binds strongly to both hGHR and hPRLR, as does hGH itself (68, 69, 70, 71). Principal human pathologies where hyperactivation of hGH-regulated signaling cascades leads to detrimental prognoses are acromegaly and cancer. The possibility of inhibiting both hGHR and hPRLR allows for a putatively improved treatment outcome in acromegaly, especially in female patients with pronounced effects of hyperprolactinemia. On the other hand, in breast cancer, as we show here (Fig. S7), patients overexpressing both hGHR and hPRLR have a significantly higher probability of mortality than patients with upregulation in either one of the receptors. Therefore, a dual antagonist of the full spectrum of hGH action, like compound G, is expected to be highly efficacious in multiple therapeutic areas (20, 72, 73). In fact, targeted hPRLR inhibition in breast cancer treatment by small molecules and monoclonal antibodies are under active development (58, 61, 74). Here, our *in vitro* assessments with human melanoma and breast cancer cells exhibit a distinct anticancer property of compound G, especially in sensitizing cells to chemotherapy. Further *in vivo* studies using xenograft or syngeneic mouse models will highlight the *in vivo* efficacy of such a treatment regimen.

In summary, a successful preparation of pegylated hGHR antagonists with markedly higher binding affinities than the currently approved hGHR antagonist, pegvisomant, is reported (Table S2). In light of the pleiotropic action of hGH, especially in later life, in driving several age-associated comorbidities including cancer, insulin resistance, cardiac hypertrophy, renal insufficiency, and tissue fibrosis, differential antagonism of hGH action is highly promising (23, 75). The congenital GHRKO mouse is the longest-lived laboratory mouse in the world but has a short stature (9). Recent reports of adult-onset ablation of GH action in fully grown mice have resulted in marked increase of lifespan, improved insulin sensitivity, decreased glomerulosclerosis, and cancer incidence in a sex-specific manner (13). Therefore, an adult life pharmaceutical intervention to reduce hGH action has been suggested (1). Herein, we provide an opportunity of such an intervention in future with a repertoire of strong preclinical candidates. Extensive pharmacologic developmental studies will identify the best molecule for future use.

## Experimental procedures

The pegylated hGHR antagonists used in the *in vitro* cell studies (compound G and compound D) and the nonpegylated hGHR antagonist hGH-G120K were prepared by first expressing a His-tagged protein with a TEV protease cleavage site. After affinity purification, removal of the His-tag, and pegylation, the final products contained five additional amino acids on their *N* termini (GGSSG). The hGHR antagonist that was prepared in larger quantities for the subsequent *in vivo* studies (compound G′) was expressed without a His-tag and contains only a single additional methionine (M) on its N terminus.

The structures of the two maleimide-activated PEG molecules used in this study (dPEGA and GL2-400 MA) are shown in Fig. S1. These molecules are conjugated to the thiol groups of cysteine molecules in the hGHR antagonist proteins by well-characterized chemical reactions (76). Table 1 shows the three pegylated hGHR antagonists prepared and tested in this study along with their abbreviations. The X-ray structure of hGH bound to the hGHR dimer (25, 26) is shown in Figure 1*A*. This structure was used as a model for the binding of hGH-G120K to the receptor (31), and the amino acid positions chosen for substitution with cysteine are shown in Figure 1*B*. The primary amino acid structures of pegvisomant, hGH-G120K, and the hGHR antagonists (compound D and compound G) described in this study, along with the positions of the native disulfide bonds and the positions of the PEG substitutions, are shown in cartoon form in Fig. S2.

### Preparation of pegylated hGHR antagonists from precursors containing N-terminal His-tags and TEV protease cleavage sites (compounds G and D)

The pegylated hGHR antagonists compound G and compound D were prepared by substituting either two or one amino acid, respectively, of the GHR antagonist GGSSG-hGH-G120K with cysteine and conjugating the added cysteines to different PEG molecules. The precursor molecules were expressed with an *N*-terminal His-tag to facilitate purification and a TEV cleavage site to enable removal of the His-tag.

#### Preparation of compound G

##### Gene construction for His-tagged hGH mutants

The WT hGH gene (somatotropin) (UniProt: UniProtKB - P01241) with an *N*-terminal hexa-histidine tag and a TEV cleavage site (His-TEV-hGH) was synthesized and cloned into pET-21 (a vector by Genscript). The His-TEV-hGH mutant was made by site-specific mutagenesis (QuickChange II site-directed mutagenesis kit; Agilent #200522) to substitute a lysine for the glycine at position 120 and to substitute a cysteine for amino acids codons at positions T142 and H151. The construct was transformed into Lemos (DE3) (New England Biolabs) for protein expression. The complete amino acid sequence for the His-tagged construct of the compound G precursor is shown below. The cysteines that are conjugated to dPEGA and the amino acid position responsible for converting hGH into a hGH antagonist (G120K) are highlighted and shown in bold:

MAHHHHHHGSSGENLYFQGGSSGFPTIPLSRLFDNAMLRAHRLHQLAFDTYQEFEEAYIPKEQKYSFLQNPQTSLCFSESIPTPSNREETQQKSNLELLRISLLLIQSWLEPVQFLRSVFANSLVYGASDSNVYDLLKDLEE**K**IQTLMGRLEDGSPRTGQIFKQ**C**YSKFDTNS**C**NDDALLKNYGLLYCFRKDMDKVETFLRIVQCRSVEGSCGF

The amino acid sequence after TEV cleavage is shown below.

GGSSGFPTIPLSRLFDNAMLRAHRLHQLAFDTYQEFEEAYIPKEQKYSFLQNPQTSLCFSESIPTPSNREETQQKSNLELLRISLLLIQSWLEPVQFLRSVFANSLVYGASDSNVYDLLKDLEE**K**IQTLMGRLEDGSPRTGQIFKQ**C**YSKFDTNS**C**NDDALLKNYGLLYCFRKDMDKVETFLRIVQCRSVEGSCGF.

##### Protein expression

Primary cultures were prepared by inoculating 2 ml of LB broth containing 100 μg/ml of carbenicillin with a frozen glycerol stock of *E. coli* containing the plasmid for MAHHHHHHGSSGENLYFQ-GGSSG-hGH-G120K-T142C-H151C. The inoculated broth was incubated at 37 °C for 4 h with shaking at 200 RPM. This culture was then used to inoculate 30 ml of LB broth, containing 100 μg/ml carbenicillin, and incubated overnight at 37 °C with shaking at 200 RPM. Four 2.5 L flasks, each containing 750 ml of TB broth and carbenicillin (100 μg/ml), were inoculated with 7.5 ml of the overnight culture and incubated at 37 °C until the A(600) of the culture was between 0.6 and 1.0 OD units (∼2 h 45 min). The culture was then cooled to 16 °C, made 1 mM IPTG, and incubated overnight with shaking at 200 RPM to induce expression. The culture was harvested by centrifugation at 6080 rcf for 15 min at 4 °C. Cell pellets from 250 ml aliquots of the cell culture were stored at −20 °C until purification.

##### Cell disruption

Cell pellets obtained from centrifugation of 250 ml of growth medium containing the expressed mutant were suspended in 10 ml PBS and combined with 0.05 ml of a protease inhibitor cocktail that did not contain EDTA (Sigma P8849). The solutions were cooled in an ice water mixture and sonicated (Fisher 100 sonic dismembrator, 5 mm probe tip) five times in 30 s bursts. After each sonication, the samples were cooled in the ice-water mixture until the temperature was below 4 °C. The sonicated suspensions were then centrifuged at 4 °C and 25,000*g* for 30 min and the supernatants collected and kept on ice.

##### IMAC purification of expressed protein

The sonicate supernatants were adjusted to 0.3 M sodium chloride and made 5 mM imidazole by addition of a pH 7 solution of 150 mM imidazole. The samples were then applied to a stoppered gravity flow column packed with 5 ml of Talon (Clontech) immobilized metal affinity resin (IMAC) equilibrated in 0.05 M sodium phosphate buffer, pH 7.0 containing 5 mM imidazole and 0.3 M sodium chloride. The tops of the columns were then stoppered, and the columns mixed end-over-end at RT for 30 min. The columns were allowed to drain and washed with at least five 5 ml aliquots of the equilibration buffer. The washing was continued until the A(280) nm of the eluents no longer decreased. The columns were then eluted with the pH 7 equilibration buffer containing 150 mM imidazole, and the product containing fractions were made 5 mM EDTA by addition of a 100 mM solution of disodium EDTA adjusted to pH 7.

##### TEV protease cleavage of His-Tag and reduction of non-native disulfides

The IMAC-purified mutants were concentrated by molecular filtration (Amicon Ultra-4 centrifugal 10 filter, 10 kDa; Millipore) to 2 mg/ml, and aliquots of the solutions were made 1.5 mM reduced glutathione + 0.15 mM oxidized glutathione. TEV Protease (TurboTev, Accelagen) was then added (0.04 mg protease/mg protein), and the solutions incubated for 2 h at RT followed by overnight incubation at 4 °C. The imidazole- and glutathione-containing buffers were then exchanged on SEC spin columns (Zeba 7 kDa, Millipore) for a buffer containing 0.05 M sodium phosphate, pH 7.0 and 0.3 M sodium chloride.

##### Pegylation

After removal of the glutathione and imidazole, the product was analyzed to determine whether the native disulfide bonds had formed so that only the amino acids that were replaced with cysteine could be pegylated. Pegylation was performed in 0.05 M sodium phosphate, pH 7.0 and 0.3 M sodium chloride. Small aliquots of the solution were made 1 mM Mal-dPEG-A and incubated for 1 h at RT before being analyzed by SDS-PAGE. In cases when the native cysteines were not fully oxidized, the gels showed bands with lower mobilities (higher MW) than the mobility of compound G, caused by pegylating both the native and the inserted cysteines. If the gel showed the product and no higher molecular weight species, then large scale pegylation was performed. If higher molecular weight bands appeared, then the formation of the native disulfides was catalyzed by the addition of divalent copper. Copper oxidation was performed by adjusting the product solution to 2.5 μM copper sulfate and incubating for 10 min. This sample was then analyzed again by SDS-PAGE to ensure that the native disulfide bonds had formed. The non-native cysteines in the compound G precursor were pegylated in the large-scale reaction by exchanging the buffer to the pH 7 phosphate buffer solution with 0.5 mM maleimide-dPEGA and incubating the reactions for 2 h at RT followed by overnight incubation at 4 °C.

##### Purification

The pegylation reaction mixture, which contained compound G, was applied to gravity flow IMAC columns containing 5 ml Talon resin equilibrated in a 0.05 M sodium phosphate buffer, pH 7.0, 0.3 M sodium chloride, and the columns were washed with 5 column volumes (CVs) of the same buffer. Compound G, which did not bind to the column, was found in the Talon flow throughs and washes, which were then concentrated on a centrifugal concentrator to 0.3 ml and purified by SEC on a Superdex 200 Increase 10/300 Gl column (GE Healthcare) equilibrated in 0.05 M Tris Buffer, pH 8, containing 0.15 M sodium chloride and 10% glycerol. The product fractions were combined and analyzed for protein concentration by absorption at A(280) nm and for purity by SDS-PAGE.

#### Preparation of compound D

The gene construction of the compound D precursor was the same as that shown for compound G, except that for compound D, only one amino acid (T142) was changed to cysteine. Protein expression, cell disruption, IMAC purification, and TEV cleavage were performed as described for compound G. Pegylation was performed with GL2-400 MA using the procedure described for compound G.

##### Purification of compound D

Compound D was purified, after pegylation and passage through the second IMAC column, by anion exchange chromatography (HiTrap Q FF, 5 ml, Cytiva). To lower the salt concentrations prior to application to the column, samples were diluted 10× with 5 mM Tris buffer, pH 8, 10% glycerol. The samples were concentrated to their original volumes on molecular concentrators and then diluted 10× a second time with the same buffer. The samples were then applied to the HiTrap Q columns equilibrated in the 5 mM Tris, pH 8, 10% glycerol buffer, and the columns were washed with 5 mM Tris, pH 8, 10% glycerol and eluted with a salt gradient from 0 to 100 mM NaCl in the same Tris buffer.

### Preparation of a pegylated GHR antagonist expressed without a His-tag (compound G′)

This section describes the preparation of compound-G’ (M-hGH-G120K-T142C- dPEG-A-H151C-dPEG-A), which was expressed and purified without a His-Tag.

#### Gene construction for an hGH mutant without a His-tag

The gene for M-hGH-G120K-T142C-H151C was synthesized and cloned into the pET-21a vector by Genscript. The complete amino acid sequence of this molecule is shown below. The construct was transformed into Lemos (DE3) (New England Biolabs) for protein expression. The three site-specific substitutions (G120K, T142C, and H151C) are highlighted and shown in bold:

MFPTIPLSRLFDNAMLRAHRLHQLAFDTYQEFEEAYIPKEQKYSFLQNPQTSLCFSESIPTPSNREETQQKSNLELLRISLLLIQSWLEPVQFLRSVFANSLVYGASDSNVYDLLKDLEE**K**IQTLMGRLEDGSPRTGQIFKQ**C**YSKFDTNS**C**NDDALLKNYGLLYCFRKDMDKVETFLRIVQCRSVEGSCGF

#### Protein expression

A primary culture was prepared by inoculating 2 ml of LB broth containing 100 μg/ml of carbenicillin with a frozen glycerol stock of *E. coli* containing the M-hGH-G120K-T142C-H151C plasmid. The inoculated broth was incubated at 37 °C for 4 h with shaking at 200 RPM. This culture was then used to inoculate 30 ml of LB broth, containing 100 μg/ml carbenicillin, and incubated overnight at 30 °C with shaking at 150 RPM. Four 2.5 L flasks, each containing 750 ml of TB broth and carbenicillin (100 μg/ml), were inoculated with 7.5 ml of the overnight culture and incubated for 2 h 45 min, at which time the A(600) nm of the cultures reached 0.6 to 1.0 OD. The cultures were then cooled to 16 °C, made 1 mM IPTG, and incubated overnight with shaking at 200 RPM to induce expression. The cultures were then harvested by centrifugation at 6080 rcf for 15 min at 4 °C, resulting in cell pellets that were stored at −20 °C until purification.

#### Cell disruption

Cell pellets from 1.5 L batches of culture were suspended by vortexing in 150 ml of cold PBS containing 5 mM cysteine and 0.1 ml of a protease inhibitor cocktail (Sigma P8849). The suspended cells were cooled in an ice water bath and sonicated (Fisher 100 sonic dismembrator, 5 mm probe tip) for 30 s followed by a 5-min incubation to cool the solution. The sequence of sonication followed by cooling was repeated five more times for a total of six sonication cycles. The samples were then centrifuged for 30 min at 30,600 rcf at 4 °C.

#### Ammonium sulfate precipitation

The supernatant from the centrifuged sonicate was stirred at 4 °C and made 1.3 M ammonium sulfate by the addition of cold 3 M ammonium sulfate in PBS, pH 7. After stirring for 15 min at 4 °C, the precipitate was pelleted by centrifugation at 4 °C for 30 min at 30,600 rcf.

#### Glutathione reduction

The ammonium sulfate precipitate was redissolved in 100 ml of cold PBS by gentle stirring at 4 °C for 30 min. The solution was then made 1.5 mM reduced glutathione, incubated for 2 h at RT, and concentrated to 50 ml using a 10 kDa concentrator. The concentrated sample was applied at RT to a 1 L Sephadex G-25 column that was equilibrated with aerated PBS to remove the glutathione and allow oxidation of the native cysteines of the M-hGH-G120K mutant to form the native disulfide bonds.

##### Pegylation

After removal of the glutathione, the oxidation state of the native hGH disulfides was determined as described for compound G and copper sulfate oxidation was performed if needed. When the native disulfide bonds had formed, the samples, which were in pH 7.4 PBS, were adjusted to 1 mg/ml and then made 0.125 mM Mal-dPEG-A. The reaction was allowed to proceed at RT for 1 h and then stored at 4 °C prior to purification.

#### Size-exclusion chromatography

Compound G′ was concentrated on a 10 kDa centrifugal concentrator to ∼10 mg/ml and then aliquots of 6 to 9 ml were applied to a HiLoad Superdex 200 pg size exclusion column at RT (Cytiva; 26 mm × 600 mm) that was equilibrated in filter-sterilized Tris-buffered saline, pH 8 containing 10% glycerol. The SEC buffer was made just prior to use with endotoxin-free water. Fractions that contained compound G were identified by SDS-PAGE and combined.

#### Cation exchange chromatography

All buffers for cation exchange chromatography were made using endotoxin-free water. The combined SEC fractions were concentrated to ∼10 mg/ml on a 10 kDa centrifugal concentrator (Amicon Ultra 15) and then diluted to 1 mg/ml with 10 mM sodium acetate, pH 4. The sequence of concentration and dilution was repeated two additional times, and the conductivity was measured. If the conductivity was <1 mSiemens, then the sample was applied to the cation exchange column. If not, then the sequence of concentration and dilution was repeated. The product was purified and endotoxin removed using cation exchange chromatography that was performed at 4 °C. Five 5-mL HiTrap SP Sepharose FF cation exchange columns (Cytiva) were connected and equilibrated in 10 mM sodium acetate, pH 4. The product, which was in a low salt pH 4 buffer, was applied to the column that was then washed with the following sequence of buffers at 4 °C: 5 CV 10 mM sodium acetate, pH 4; 30 CV 0.1% Triton X-114 in 10 mM sodium acetate, pH 4; 10 CV 1% CHAPS in 10 mM sodium acetate, pH 4; and 5 CV 10 mM sodium acetate, pH 4. The column was then eluted stepwise with a 10 mM sodium acetate buffer (pH 4) containing different concentrations of sodium chloride; 20 CV 100 mM sodium chloride, 10 CV 300 mM sodium chloride, and 10 CV of 500 mM sodium chloride. Fractions (2 ml) were collected in tubes that contained 0.2 ml 1 M potassium phosphate, pH 8.5 to adjust the pH of the eluted fractions to neutrality. The majority of the product eluted in the buffer containing 300 mM NaCl. Fractions that contained pure product were identified by SDS-PAGE. These fractions were collected, and the buffer was exchanged for PBS using the concentration/dilution procedure described above. The final product was found to contain <1 EU endotoxin/mg protein using the Pierce Chromogenic Endotoxin Quantitation Kit (S39552).

### Characterization of the pegylated GHR antagonists

#### SDS-PAGE, analytical SEC, and MALDI-TOF

SDS-PAGE was performed with 4%-20% gradient gels (Bio-Rad 4561093) using a 1X Tris/Glycine/SDS buffer (Bio-Rad 1610732). Samples were heated at 95% C for 5 min in 1X Laemmli buffer (Bio-Rad 1610737) under nonreducing condition. SEC was performed on a Superdex 200 Increase column, 10 × 300 mM (Cytiva 28990944) on an Agilent 1200 HPLC system. The flow rate was 0.75 ml/min and the buffer was PBS. Mass spectrometry was performed on a Bruker Ultraflextreme MALDI TOF-TOF.

#### GHR antagonist stability

The hGHR antagonists and hGH in pH 7.4 PBS were tested for freeze-thaw stability by rapidly thawing small aliquots (10 μl) of the GHR antagonists in a RT water bath, incubating the aliquots on ice for 2 h, and then refreezing the samples in a −80 °C freezer overnight. One, two, and three freeze-thaw cycles were performed. “Room Temperature” stability was determined by incubating samples for 2 h, 4 h, and overnight (25 h) in a 24 °C incubator. The stability of the samples at 4 °C was determined by incubating the samples for 2 h, 4 h, and overnight in a refrigerator. At both temperatures, the 2 h and 4-h samples were frozen, and the three time points assayed together the next day. Competitive binding assays were performed at 17 nM GHR antagonist, and the percent activity retained after freeze-thaw cycles or incubation at 4 °C or 24 °C were determined from the assay response of the starting sample divided by the assay response of the treated sample.

### Receptor-binding assays

#### Preparation of biotin-hGH

hGH (Thermo RP-10928) was dissolved at 1 mg/ml in a 0.025 M sodium phosphate buffer at pH 7 and purified from any small molecule contaminants on a 2 mL Zeba SEC spin column (Thermo Fisher Scientific, 89890) equilibrated in the same buffer. The solution was then made 0.25 mM Biotin-dPEG4-TFP (Quanta BioDesign 10009) and incubated for 1 h at RT. The reaction product was then purified on a second 2 mL Zeba spin column equilibrated in PBS and frozen in single use aliquots at −80 °C.

#### Affinity for the hGH receptor

The relative affinities of hGH and the different pegylated GHR antagonists for the hGHR were compared in competitive ELISA assays. To develop this assay, it was necessary to determine the concentration of biotin-hGH that would be included in every well. Soluble, recombinant GHR (R&D Systems 1210-GR) was coated on an ELISA plate at 0.125 μg/ml in PBS for 1 h at 37 °C, and after washing with PBS/0.05% Tween 20 and blocking with 2% bovine serum albumin in PBS, the wells were incubated with different concentrations of biotin-hGH. After washing, treatment with 0.5 μg/ml streptavidin-horse radish peroxidase (Thermo Fisher Scientific, 21130), washing, incubation with 3,3′,5,5′-tetramethylbenzidine (SeraCare 5120-0083), and addition of 1 M HCL, the concentration of biotin-hGH that gave an A(450 nm) assay response of approximately 2 was selected. The selected biotin-hGH concentration (0.1 μg/ml) was then incubated with different concentrations of hGH or the pegylated GHR antagonists, and the abilities of these compounds to inhibit the binding of biotin-hGH to the plate were determined in the assay described above. The IC50 values from these assays, determined graphically (Table 2), reflect the relative affinities of hGH and the pegylated GHR antagonists for the GHR.

#### Affinity for the hPRL receptor

The relative affinities of hGH and the hGHR antagonists for the hPRLR was done using the same procedure described above for the hGHR except that the plate was coated with 0.3 μg/ml of the recombinant hPRLR (R&D Systems 1167-PR), and the inhibition assays were performed in the presence of 0.5 μg/ml biotin-hGH.

### Biological assays for GHR inhibition

The abilities of the purified and pegylated compounds to inhibit hGHR activation was assessed using a surrogate assay, tyrosine phosphorylation of STAT5, as hGH binding to hGHR is followed by immediate activation of hGHR-associated JAK2 kinase which in turn tyrosine phosphorylates STAT5 (77).

#### Cell culture and hGH treatments

The human melanoma cell line MALME-3M was used for most of the assays, in addition to SK-Mel-28 (human melanoma), SK-Mel-30 (human melanoma), MDA-MB-231 (human breast cancer), T47D (human breast cancer), MB49 (mouse bladder cancer), and L cells (mouse fibroblast). MALME-3M has relatively high levels of GHR (Fig. S6). Cells were cultured in either Eagle's Minimum Essential Medium (EMEM), Roswell Park Memorial Institute 1640, IMDM, or Dulbecco's Modified Eagle Medium (ATCC) supplemented with 10% fetal bovine serum (FBS) and antibiotics (penicillin-streptomycin) as per manufacturer’s instructions. Cells were grown at 37 °C under a 5% CO_2_ atmosphere in a humidified incubator, and growth media was replaced every 48 h during incubation. No hGH was present in or added to the growth media unless specifically indicated. After seeding in 6-well plates at a concentration of 300,000 cells/ml overnight, cells were switched to serum-free growth media (no GH) and starved for 4 h before the addition of hGH (to human cells) or bovine (b)GH (to mouse cells) at the concentrations noted. Cells were then allowed to incubate for an additional 15 min before being harvested.

#### Protein extraction

Following treatment, cells were harvested at the indicated time points and total protein was extracted. To promote protein extraction, the treatment media was removed by aspiration and the cells were washed twice with ice-cold PBS. Following washing, total protein was extracted from the cells using RIPA buffer (100 μl/million cells) supplemented with 1.5× Halt protease and phosphatase inhibitor cocktail. Briefly, chilled RIPA buffer was added to the cells and was allowed to incubate for 5 min at 4 °C. Adherent cells were harvested for lysis using a sterile cell scraper. The resultant cell suspensions were sonicated on ice for 5 min with ON (2-s)/OFF (1-s) pulses at 2% power. The cell lysates were then cleared by centrifugation at 8000*g* for 10 min at 4 °C. Following centrifugation, the supernatants were collected and stored at −80 °C until further use. Sample protein concentrations were determined using a Bradford assay with bovine serum albumin as the protein standard. Absorbances at 595 nm were measured using a Spectramax 250 multi-mode plate reader (Molecular Devices) and processed using Softmax Pro v4.7.1 software (https://support.moleculardevices.com/s/article/SoftMax-Pro-7-1-software-Download-page).

#### Western blot

Cytosolic fractions from treated cells were thawed on ice, and 100 μg protein was loaded onto polyacrylamide gels for separation by SDS-PAGE. All SDS-PAGE gels were resolved at 120 V for 1.5 h at 20 °C. Following separation, the proteins were transferred to activated PVDF membranes using a wet-transfer method at 20 V for 16 h at 4 °C. The membranes were then blocked with 5% nonfat dry milk in 1× Tris buffered saline, 0.1% Triton-X 100, pH 7.2 for 1 h at 25 °C and incubated with the primary antibody (rabbit anti-STAT5A/B; Biotechne) at specified dilutions for 16 h at 4 °C. Following addition of the primary antibody, the membranes were washed and subsequently incubated with appropriate secondary antibodies (Goat anti-Rabbit horse radish peroxidase; Cell Signaling Technology) at the indicated dilutions for 2 h at 25 °C. Membranes were then washed with Tris buffered saline, 0.1% Triton-X100 and treated with West Femto Chemiluminescence detection reagents (Thermo Fisher Scientific). Chemiluminescence signals were detected using an Odyssey XF (LI-COR) imaging system. Densitometry analyses of the blots were performed using ImageJ Software (https://imagej.nih.gov/ij/) by measuring the integrated band density and normalizing against band intensity of the loading control (b-actin) in the same lane. The integrated densities of pSTAT5 bands from cells after different treatments were then normalized to integrated densities of pSTAT5 bands from cells treated with hGH alone to obtain an induction ratio. Percent inhibition was then measured by subtracting the pSTAT5 band intensity in each lane from the pSTAT5 band intensity of hGH-treated samples.

### Assays for anticancer properties of hGHR antagonist compound G

#### Crystal violet cell viability assay

This assay was performed to assess the number of cells with colony-forming capacity, following hGHR antagonist and/or chemotherapy treatment. Human melanoma cells cultured in 10% FBS and 1X Pen-Strep containing EMEM media were plated onto 12-well plates at a concentration of 200 cells/cm^2^ and, following cell-attachment (overnight), they were treated with either saline or recombinant hGH (@2.5 nM) or the chemotherapy doxorubicin (@200 nM) or both GH and doxorubicin for 72 h. After 72 h, cells were stained with crystal violet staining solution (Abcam, cat# ab232855) for 15 to 20-min, washed, air dried, photographed, and then solubilized, and the absorbance was measured at 570 nm using Spectramax 250 (Molecular Devices) spectrophotometer with a Softmax Pro v4.7.1 software.

#### Resazurin cell viability assay

This assay was performed to quantify the effect of hGHR antagonist and/or chemotherapy treatments on cell viability. Human melanoma cells cultured in 10% FBS and 1X Pen-Strep containing EMEM media were plated in 96-well plates at a concentration of 10,000 cells/well, and following cell-attachment (overnight), they were treated with 2% FBS and 1X Pen-Strep containing EMEM media with either saline or recombinant hGH (@5 nM) or the GHR antagonist (@500 nM) or both hGH and hGHR antagonist for 72 h. After 72 h, the media was removed and Resazurin reagent (Abcam, cat# 129732) was added, incubated for 4-h, and the absorbance was measured at 570 nm (reference wavelength 600 nm) using a Spectramax 250 (Molecular Devices) spectrophotometer with Softmax Pro v4.7.1 software.

#### Colony formation assay

This assay was performed to assess the number of cells in the supernatant, following treatments (hGHR antagonist or chemotherapy or both) with colony-forming capacity. MALME-3M cultured in 10% FBS and 1X Pen-Strep containing EMEM media were plated onto 6-well plates at a concentration of 200,000 cells/well and treated with 200 nM doxorubicin for 72 h. After 72 h, the supernatant (2 mL) was collected (only to assess detached but viable cells) and 200 μL of it was plated on to 24-well plates in 500-μL 10% FBS and 1X Pen-Strep containing EMEM media and incubated for 14 days. After 14 days, the media was removed and cells were washed and stained with crystal violet staining solution (Abcam, cat# ab232855) for 15 to 20 min and then washed, air dried, photographed.

#### Cell migration assay

This assay was performed to assess the effect of hGHR antagonist on hGH-induced migration property of the cancer cells. The Radius Cell Migration Assay kit from Cell Biolabs (Cell Biolabs #CBA-125) was used for migration assay, and experiments were performed as per manufacturer’s protocol. Briefly, the assay was performed using 24-well plates containing a nontoxic, 0.68 mm biocompatible hydrogel spot that is present at the center of the well, where cells cannot attach. Prior to plating, cells were treated for 48 h, trypsinized, counted, and seeded at 5000 cells/well in the pretreated hydrogel spot containing 24-well plate. The hydrogel spot was gently removed after 24 h incubation at 37 °C/5% CO2, and cells were allowed to migrate for up to 48 h while images were captured every 24 h using a 4× objective (total magnification 40×) employing an inverted Olympus IX70 microscope fitted with a Retiga 1300 camera (QImaging). The total uncovered area at the beginning and end of assay were quantitated using ImageJ software. Experiments were done in triplicates.

#### Cell invasion assay

This assay was performed to assess the effect of hGHR antagonist on hGH-induced invasion property of the cancer cells. The cells were pretreated with GH in 2% FBS-containing Dulbecco's Modified Eagle Medium or EMEM for 48 h. The CytoSelect 96-well Cell Invasion Assay kit (CBA-112, Cell Biolabs, INC) was used as per the manufacturer’s instructions. Briefly, on the day of the assay, the cells were trypsinized, counted, and 100,000 thousand cells were then seeded per well in the upper chamber of the 96-well invasion assay well coated with basement membrane and incubated with respective treatments in serum-free media for 24 h. After 24 h, the cells from under the membranes were dislodged, lysed, and stained with CyQuant GR dye solution. The fluorescence intensity correlating with invasive cell number was measured at ex-480 nm/em-520 nm using a fluorescence plate reader.

#### Drug retention assay

This assay was performed to assess the effect of hGHR antagonist on hGH-induced drug efflux property of the cancer cells. MALME-3M cells were pretreated with 2.5 nM hGH or 2.5 nM hGH + 500 nM hGHR antagonist in 2% FBS-containing IMDM media for a week with the media being refreshed every 48 h. On the assay day, cells were trypsinized, counted, and suspended at 1 million cells per ml in cold DiOC_2_ (3) dye on ice for 15 min (Chemicon International, #ECM910). The cells were then centrifuged, and the supernatant removed. The cell pellets were subsequently resuspended in cold efflux buffer and distributed into different Eppendorf tubes under the following conditions: one set of tubes was kept on ice to deactivate the drug-efflux pumps as controls, while the other two sets were kept in a 37 °C water bath for 30 min and 120 min, respectively, allowing the active drug-efflux pumps to drive out the DiOC_2_ (3) dye. Afterward, the cells were centrifuged and washed. Cell suspensions were then dispensed into the wells of a black-walled 96-well plate, and the fluorescence was measured in a fluorescence plate reader at an excitation wavelength of 485 nm and an emission wavelength of 530 nm.

### *In vivo* efficacy of hGHR antagonist compound G’

Nude mice (Jackson Laboratories) animals were purchased at the age of 6 to 8 weeks and maintained in sterile cages with *ad libitum* access to autoclaved water and feed (ProLab RMH 3000; 14% of energy from fat, 60% from carbohydrates, and 26% from protein; PMI Nutrition International). The cages were maintained at 22 °C in a humidity-controlled room and exposed to a 14-h light, 10-h dark cycle. Twelve-week-old male Nude mice were injected intra-peritoneally (i.p.) with 100 or 200 μg/g body weight of GHR antagonists as outlined in the Results section. Body weight was measured using a Mettler Toledo PL 202-S balance. Body composition was measured using the Minispec mq Benchtop NMR analyzer (Bruker Instruments, Minispec ND2506). There were six mice in each treatment group.

#### Assays for circulating IGF1 and IGFBP3

Blood collection at specified time points was performed by placing the mice in Plexiglass restrainers, snipping <1 mm from the tip of the tail with sharp surgical scissors wiped with 70% alcohol and kneading the tail gently to obtain 200 μl of blood collected in a heparinized capillary tube. The end-of-life blood collection was done just before euthanasia by retro-orbital bleeding. All procedures performed were approved by the Ohio University IACUC. IGF1 levels were measured using an IGF1 mouse/rat ELISA kit (22-IG1MS-E01; ALPCO), and IGFBP3 levels were measured using an IGFBP3 mouse ELISA kit (EMIGFBP3; Invitrogen).

### Statistics

All values for the compound characterization assays are given as mean ± SD. Statistics were performed using GraphPad Prism software (v7.04; https://www.graphpad.com/features). For all *in vitro* biological assays, Tukey’s Multiple Comparisons test was performed while for all *in vivo* experimental comparisons, Bonferroni’s Multiple Comparison’s test was performed and corresponding scatter-plots with bars are presented. Differences were statistically significant when *p* < 0.01.

## Data availability

All data described in the manuscript are contained in the main text or supporting information. The proprietary materials used in this manuscript can be made available to other investigators at payments to cover the costs of production and reagents. All requests for protocols and proprietary material distribution should be addressed to both Nicholas Henderson (nhenderson@infinixbio.com) and John J Kopchick (kopchick@ohio.edu).

## Supporting information

This article contains supporting information.

## Conflict of interest

R. Ba., R. Br., U. S., and J. J. K. hold patent rights to the novel pegylated GHR antagonists designed and discussed in this manuscript. All other authors declare that they have no conflicts of interest with the contents of this article.
