# Supplementary material for: Structure and function of a dual antagonist of the human growth hormone and prolactin receptors with site-specific PEG conjugates
Source: J Biol Chem. 2023 Jul 11;299(8):105030. doi: 10.1016/j.jbc.2023.105030 (PMC10410519; doi:10.1016/j.jbc.2023.105030)

Supplementary Figures

(A) Maleimide-dPEGA

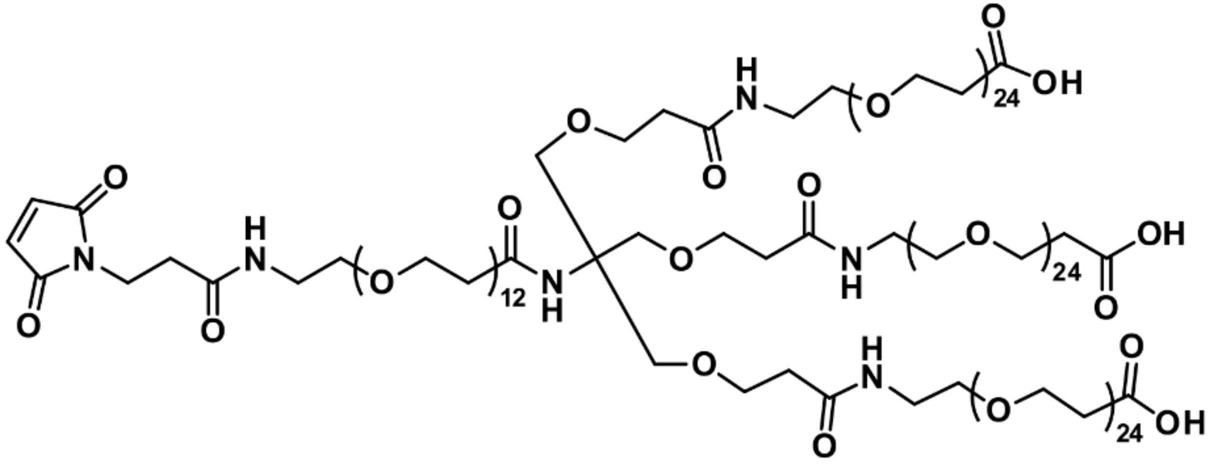

(B) GL2-400MA

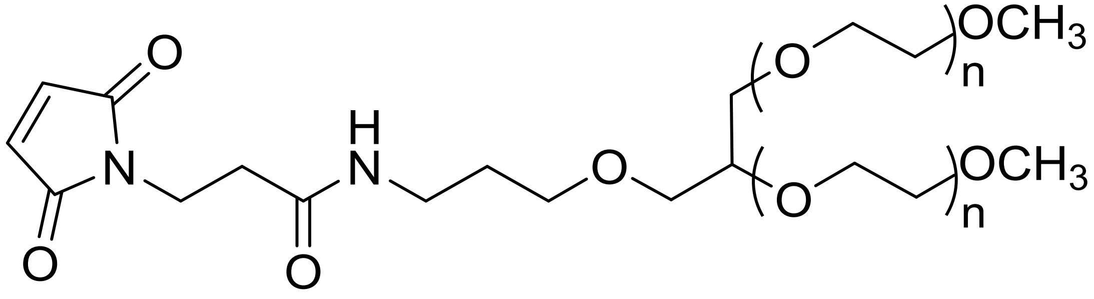

Supplementary Figure 1: Structures of the different poly-ethylene-glycols used in this study. A. Maleimide-dPEGA: MAL-dPEG®$_{12}$-Tris(-dPEG®$_{24}$-acid)$_3$, MW = 4473.2 Da., Quanta BioDesign. B. GL2-400 MA: SUNBRIGHT GL2-400MA, MW = ~ 40,000 Da, NOF America.

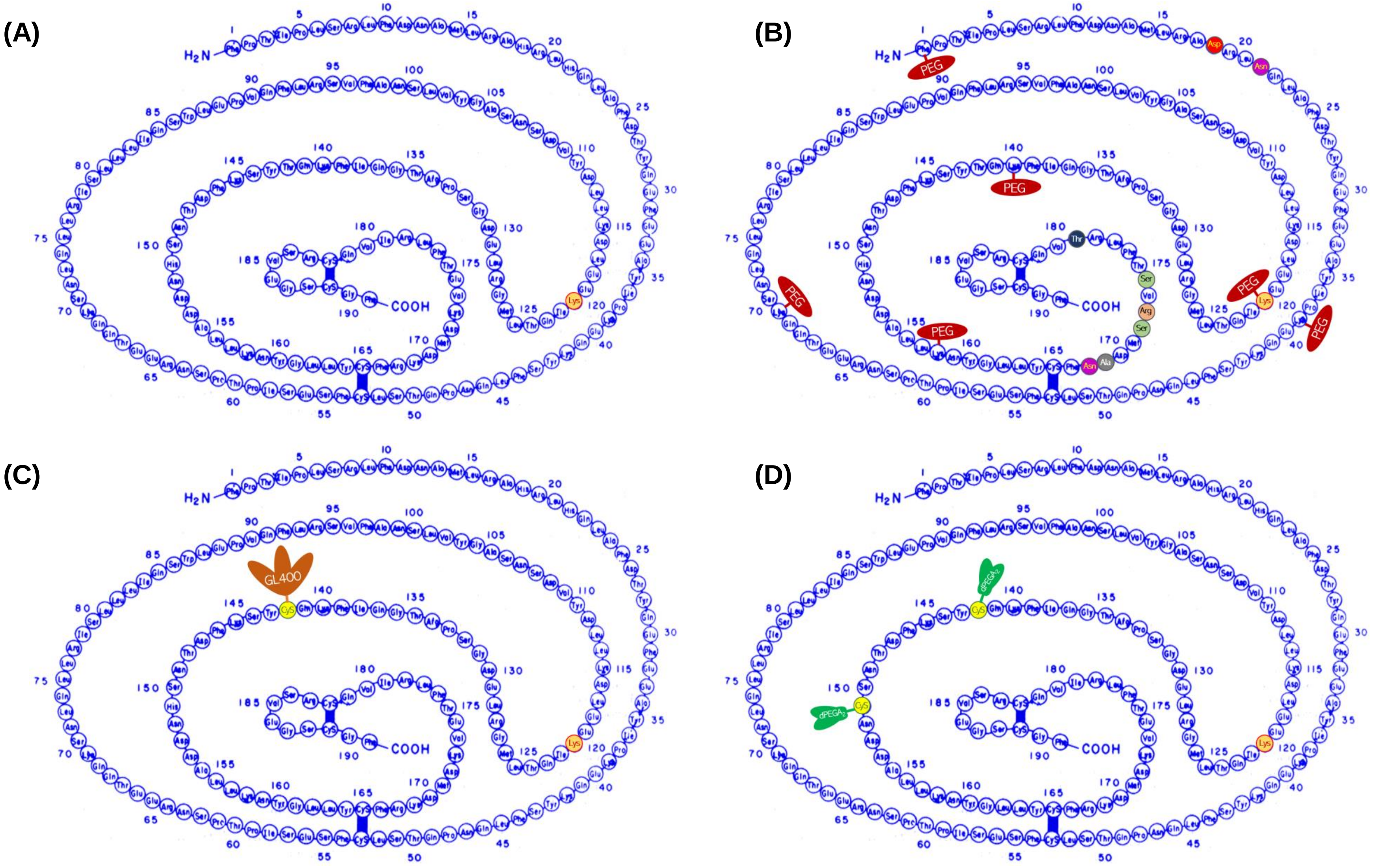

Supplementary Figure 2: Cartoons of human growth hormone receptor antagonists. (A) G120K human growth hormone (hGH), (B) Pegvisomant, (C) compound-D, (D) compound-G. The N-terminal amino acid modifications are not shown in this figure,

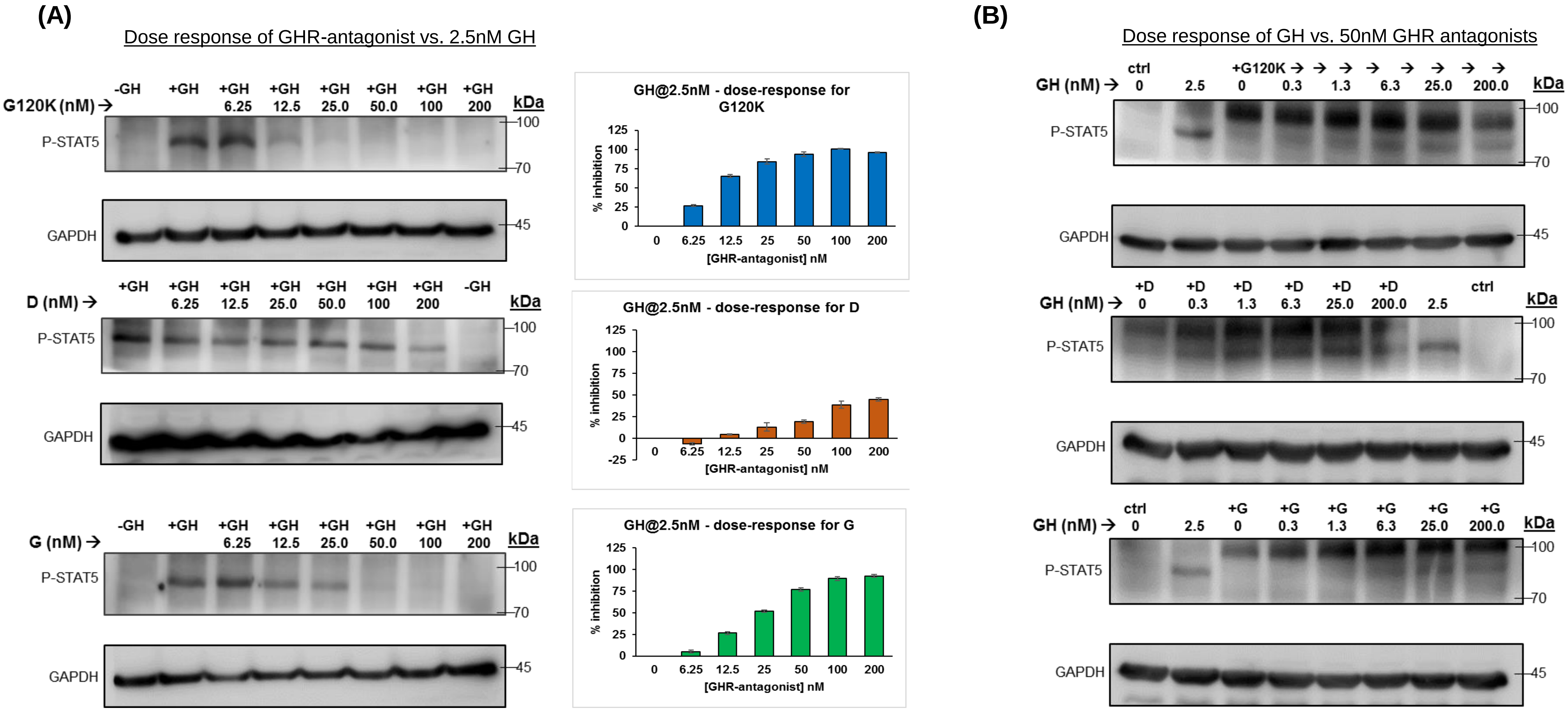

Supplementary Figure 3. Biological assay for inhibition of GHR activation by pegylated GHR antagonists. Successful binding of GH to GHR leads to downstream activation of STAT5 by phosphorylation at residues 694 and 699 by GHR-associated JAK2. This phosphorylation of STAT5 is a hallmark of GHR signaling and is used as a surrogate assay for GHR activation / inhibition. **(A)** Western blot analysis of STAT5 phosphorylation following treatment with increasing doses (6.25-200nM) of GHR antagonists vs. 2.5nM hGH. in GHR rich human melanoma cells MALME-3M. **(B)** Western blot analysis of STAT5 phosphorylation following treatment with increasing doses (0.3-200nM) of hGH vs. 50nM GHR antagonists.

[NOTE: Figure S3A is same as main Fig 2E. Fig 2E is re-used here to compare the two types of dose-response: GH vs G120K (or compound-G) in Fig-S3A against G120K (or compound-G) vs GH I nFig-S3B]

Supplementary Table 1. Stability of GHR Antagonists[1]

A. Freeze-Thaw Stability

Freeze-Thaw Cycles	hGH Run 1	hGH Run 2	Cpd G	Cpd D	Cpd G'
0	100% [2]	100% [2]	100%[2]	Not Assayed	100%[2]
1	73% ± 9%	69% ± 3%	88% ± 7%	100%[1]	101% ± 11%
2	ND	ND	90% ± 9%	84% ± 13%	107% ± 13%
3	ND	ND	99% ± 11%	84% ± 10%	89% ± 21%

B. 4°C Stability

Incubation Time (hours)	hGH 4°C	Cpd G 4°C	Cpd D 4°C	Cpd G' 4°C
0 (Fresh)	100% [2]	100% [2]	100% [2]	100% [2]
2	112 ± 29%	105 ± 13%	103 ± 14%	110 ± 21%
4	107 ± 13%	114 ± 5%	116 ± 24%	83 ± 13%
25 (ON)	106 ± 13%	97 ± 1%	117 ± 45%	89 ± 14%

C. 24°C Stability

Incubation Time (hours)	hGH 4°C	Cpd G 4°C	Cpd D 4°C	Cpd G' 4°C
0 (Fresh)	100% [2]	100% [2]	100% [2]	100% [2]
2	95 ± 13%	104 ± 7%	97 ± 10%	104 ± 1%
4	96 ± 6%	120 ± 35%	97 ± 5%	94 ± 3%
25 (ON)	93 ± 11%	131 ± 39%	89 ± 8%	94 ± 1%

[1] The stability of hGH and the GHA antagonists was calculated by dividing the assay response of the compounds before treatment (defined as 100%) by the assay response after the different treatments. All of the before treatment samples were aliquots that had been frozen at -80°C. Therefore, the before treatment sample for the freeze-thaw studies has undergone 1 Freeze-Thaw cycle.

[2.] Defined as 100%

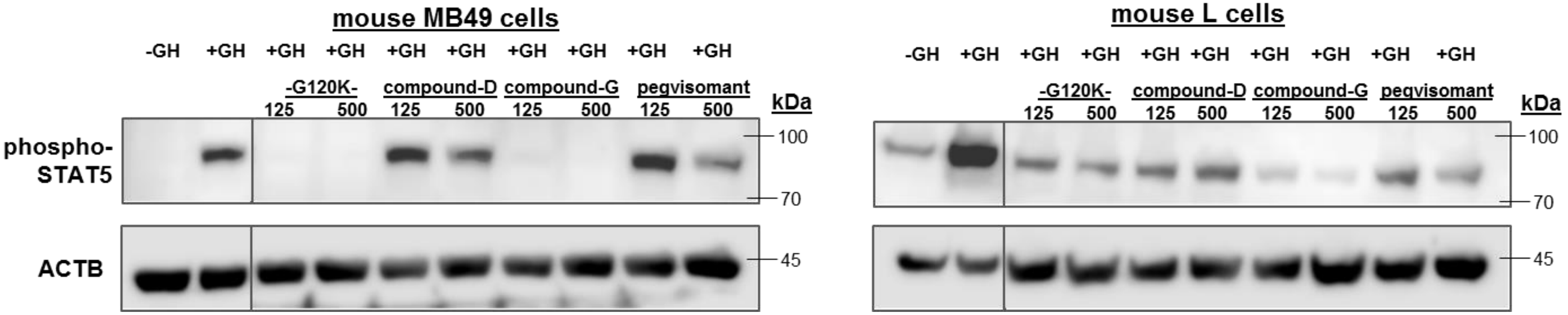

Supplementary Figure 4: **GHR antagonists (compound-D and compound-G) effectively inhibit GH signaling in mouse cells**. Western blot analysis of STAT5 phosphorylation following treatment with 12.5nM (250ng/mL) of bovine GH (bGH) and either 125nM or 500nM of GHR antagonists (either G120K, or compound-D, or compound-G, or pegvisomant) in mouse MB49 (bladder cancer) cells.

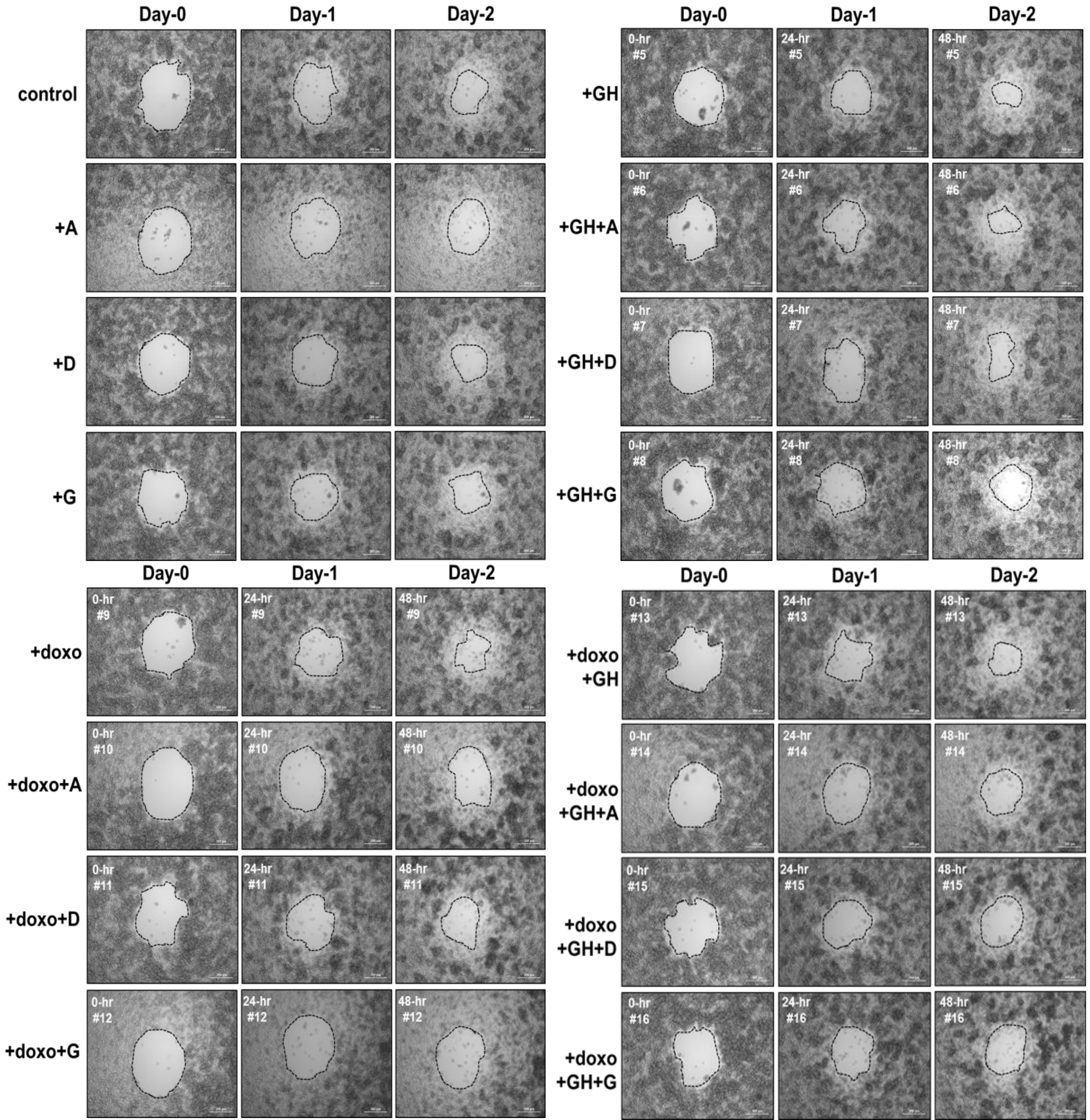

Supplementary Figure 5: Cell migration assay with human melanoma SK-Mel-28 cells treated with either PBS (control) or GH or doxorubicin or doxorubicin+GH in the presence or absence of GHR antagonist (A or D or G) for 48-hr using the Radius Cell Migration kit (Cell Biolabs). [***In each image, scale bar (bottom right) = 300 um***]

Ct values

Cell line	GHR	PRLR	GHR:PRLR ratio
SK-Mel-28	25.1	31.2	100
MALME-3M	24.0	27.9	7.2
SK-Mel-30	25.6	29.8	7.1
MDA-MB-231	26.0	25.0	0.49
T47D	21.5	16.2	0.04
H838	>30	>30	none

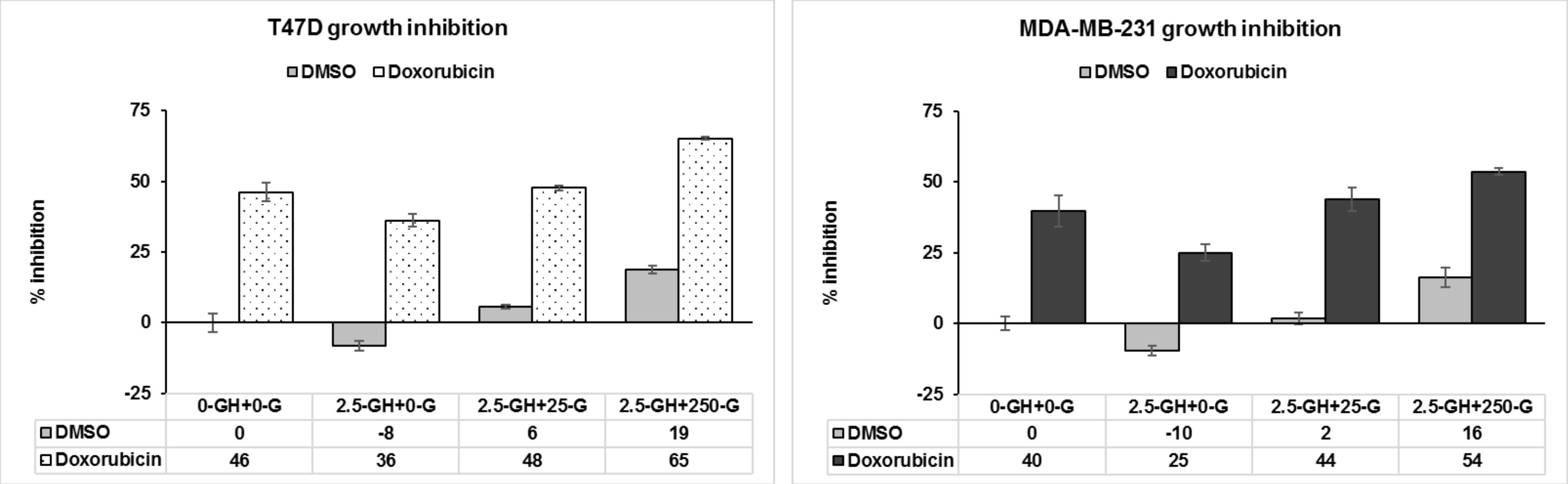

<u>Supplementary Figure 6</u>: Inhibition of growth of breast cancer cells with high PRLR expression by candidate GHR antagonists: Cell viability assay with human breast cancer cells (T47D and MDA-MB-231) treated with either DMSO (control) or 2.5nM GH or 100nM doxorubicin or doxorubicin+GH in the presence or absence of GHR antagonist compound-G (25 or 250nM) for 72-hr.

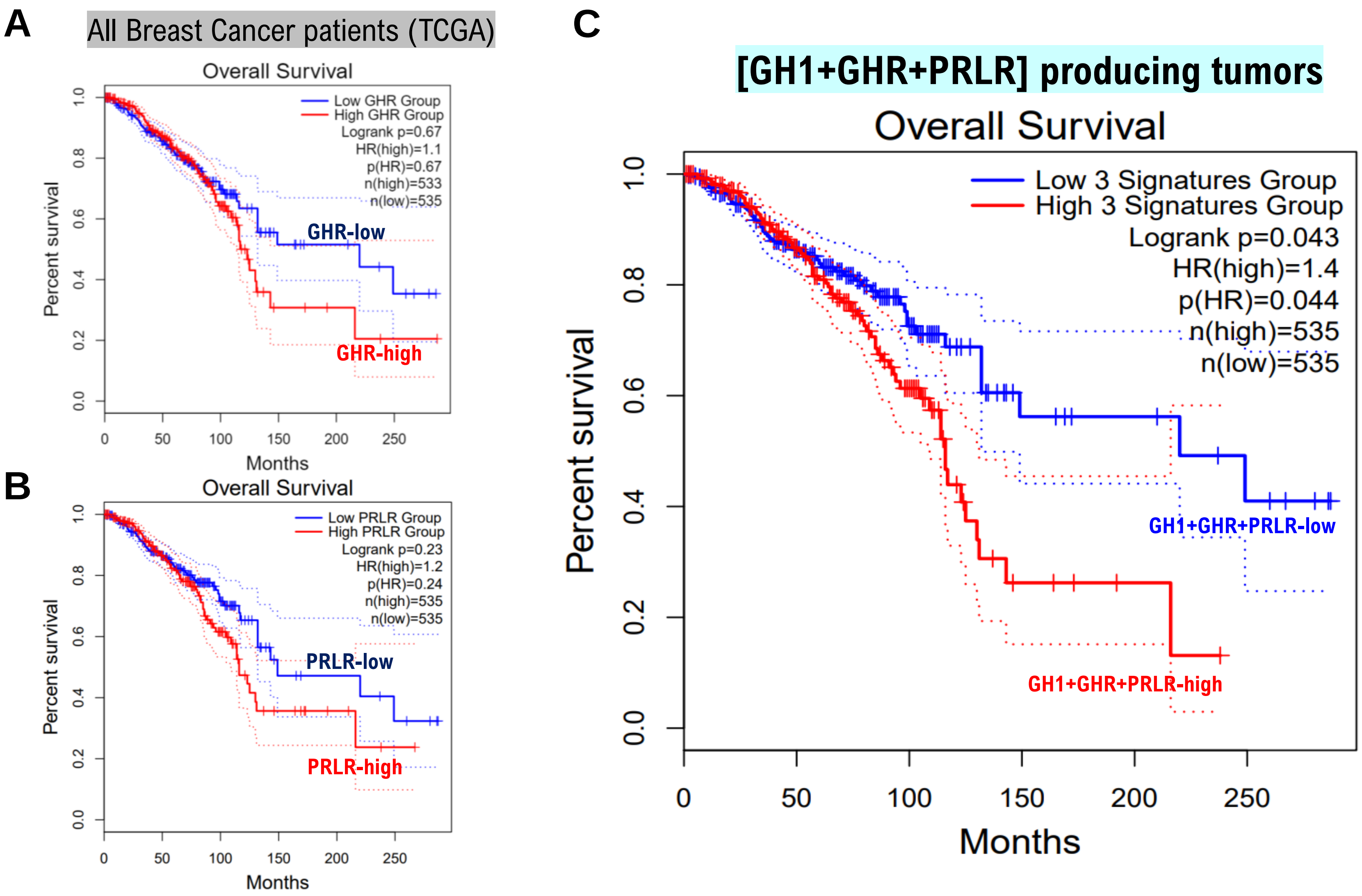

Supplementary Figure 7: Kaplan Meier survival plots for breast cancer patients (The Cancer Genome Atlas database) with high or low (<median>) tumoral expression of either GHR (A) or PRLR (B) or all of GH, GHR and PRLR (C). KM plots were generated using the GEPIA2 platform.

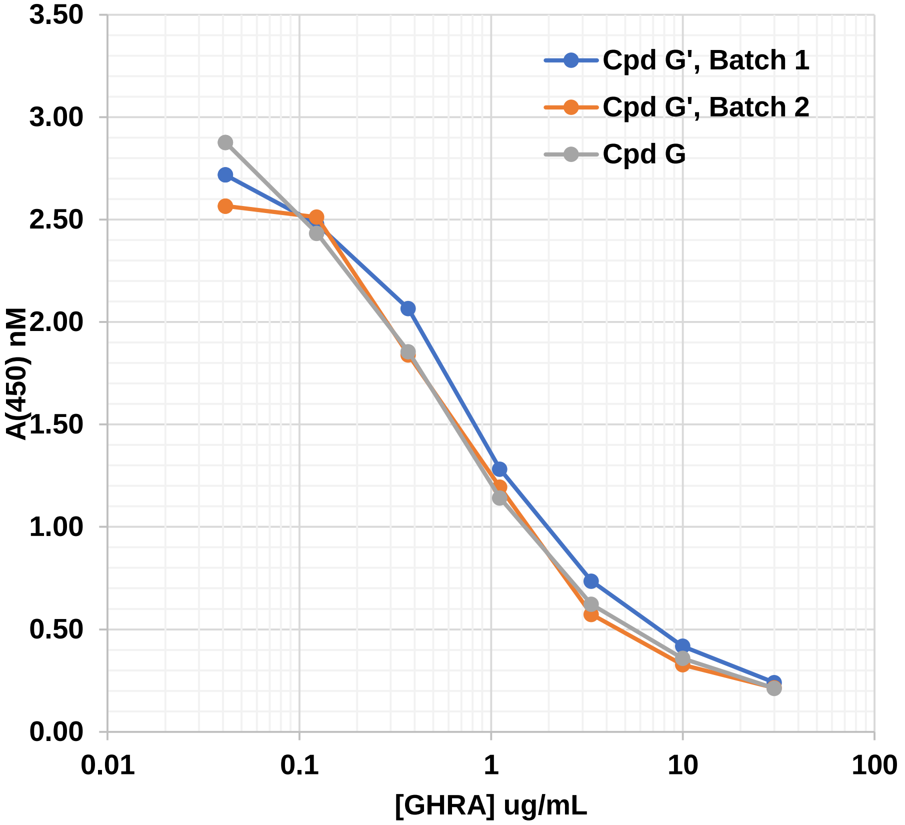

Supplementary Figure 8: Competitive ELISA for hGHR binding against Biotin-hGH of Compound G and two Batches of Compound G'. The activities of Compound-D and both batches of compound-G' are comparable and reflect batch-to-batch consistency of preparation of pegylated GHR antagonists.

properties	**Pegvisomant**	**Compound-G**
End-product heterogeneity	yes	no
Number of PEG molecules	4-6	2
Position of PEG attachment	Random	Fixed
GHR binding affinity (compared to hGH = G120K)	<=5%	75%
Phospho-STAT5 inhibition IC50	7.8nM (G120K)	21nM
Tumor inhibition *in vitro*	>100nM	>100nM
Sensitization to chemotherapy	yes	yes
PRLR binding	no	yes
Effective in GHR+ cancers?	yes	yes
Effective in PRLR+ cancers?	no	yes

Supplementary Table 2: Comparison of biological properties of pegvisomant versus compound-G

Processed Images for Main Fig 2

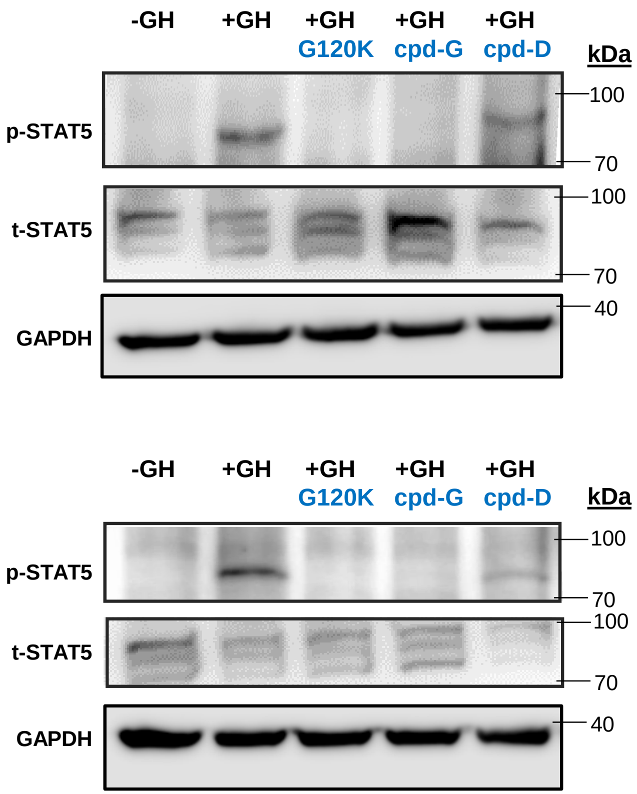

Raw Images for Main Fig 2

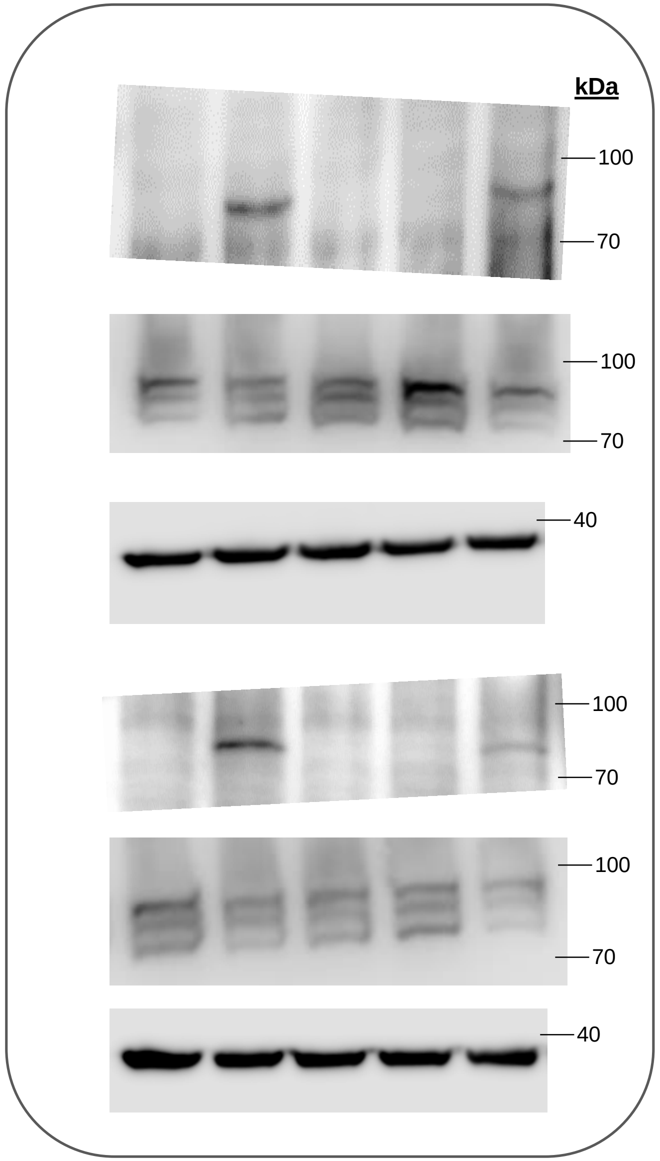

Raw Images for Main Fig 2 and Suppl. Fig 3

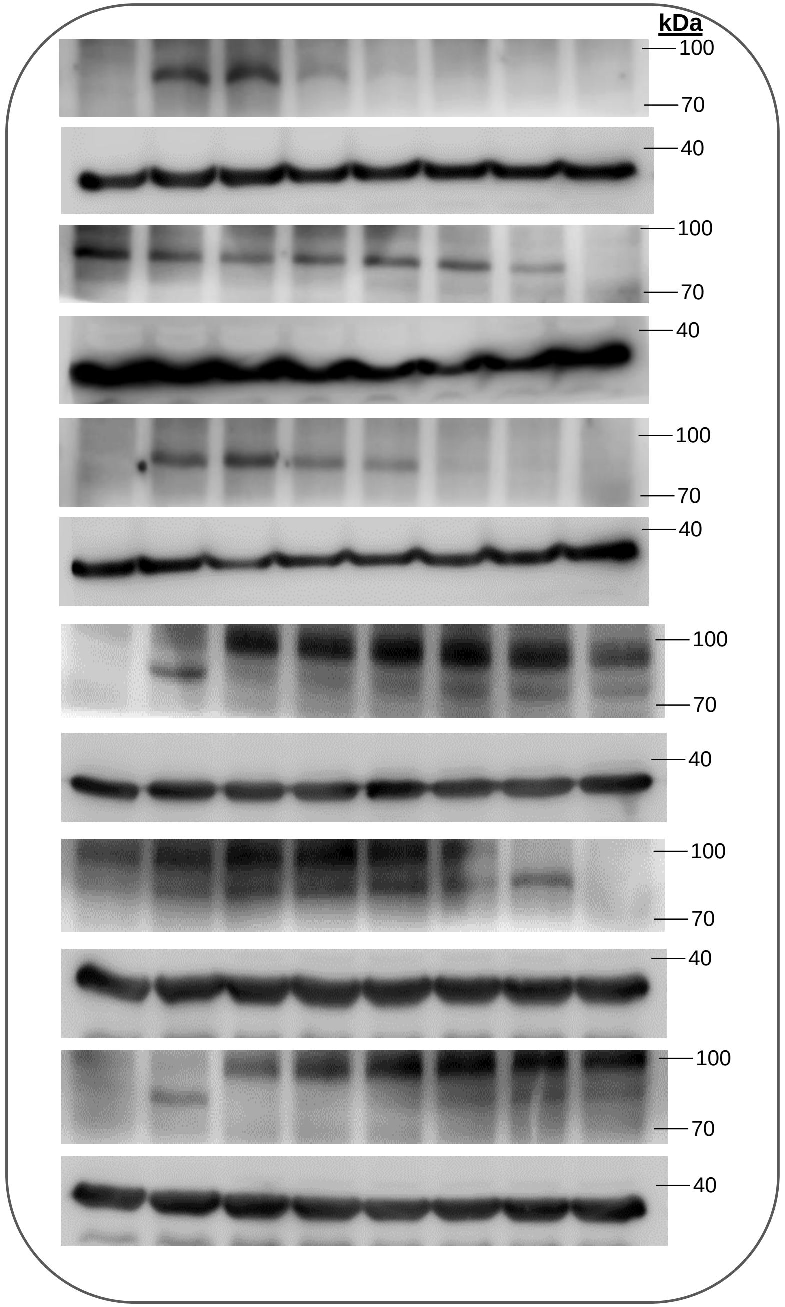

Supplement: Supporting Information [file mmc1.pdf]
